# The Yeh pilus adhesin is equipped with an α-helical flap motif, which contributes to pectin adherence

**DOI:** 10.1126/sciadv.adz1301

**Published:** 2026-01-07

**Authors:** Kevin O. Tamadonfar, Roger D. Klein, Edward D. B. Lopatto, Maxwell I. Zimmerman, Philippe N. Azimzadeh, Denise A. Sanick, Justin R. Porter, Jesus Bazan Villicana, Jerome S. Pinkner, Bo Huey Chiang, Nathaniel C. Gualberto, Karen W. Dodson, Michael L. Patnode, George M. H. Birchenough, Gregory R. Bowman, Scott J. Hultgren

**Affiliations:** ^1^ Department of Molecular Microbiology and Center for Women’s Infectious Disease Research, Washington University School of Medicine, St. Louis, MO, USA.; ^2^ Department of Biochemistry and Molecular Biophysics, Washington University School of Medicine, St. Louis, MO, USA.; ^3^ Department of Microbiology and Environmental Toxicology, University of California, Santa Cruz, Santa Cruz, CA, USA.; ^4^ Department of Medical Biochemistry & Cell Biology, Institute of Biomedicine, University of Gothenburg, Gothenburg, Sweden.; ^5^ Wallenberg Centre for Molecular & Translational Medicine, University of Gothenburg, Gothenburg, Sweden.; ^6^ Department of Biochemistry and Biophysics, University of Pennsylvania, Philadelphia, PA, USA.

## Abstract

The Yeh chaperone-usher pathway (CUP) pilus adhesin is encoded in one-half of all *Escherichia coli*. Yet little is known about its structure and function in *E. coli* persistence and pathogenesis. Structural investigations reveal that the adhesin receptor binding domains (RBDs) of YehD and its relative YhlD both share a canonical β-rich core and an α-helical flap motif that is hinged at the distal end of the core. This flap was observed in both open and closed conformations using molecular dynamics simulations. The closed conformation is dependent on a hydrophobic patch of amino acids on the distal end of the flap. Functionally, YehD_RBD_ is able to bind pectin, a polysaccharide ubiquitous in plant material. Mutations that interrupt the closed conformation increase the affinity of the protein to pectin, suggesting that the flap contributes mechanistically to pectin binding. Furthermore, in vivo, the pilus contributes to gastrointestinal (GI) tract colonization in the absence of the type 1 pilus. Hence, we report the ability of YehD to bind pectin representing a possible colonization mechanism of the GI tract via a structurally distinct CUP adhesin.

## Teaser

YehD and YhlD are α helix–containing bacterial adhesins; the former adheres to pectin and mediates bacterial GI colonization.

## INTRODUCTION

Urinary tract infections (UTIs) are primarily caused by uropathogenic *Escherichia coli* (UPEC) (*1*) and are a major driver of antibiotic usage (*2*). As antibiotic resistance increases, there is an increasing need for better understanding of the pathophysiology of UPEC infections to devise antibiotic-sparing methods of treatment. UTIs involve colonization of the bladder (cystitis) or kidneys (pyelonephritis) (*1*), and the gastrointestinal tract (GIT) can serve as a reservoir for these infections (*3*–*5*). The ability of UPEC to thrive in multiple habitats is, in part, due to their arsenal of chaperone-usher pathway (CUP) pili tipped with adhesins that determine these tropisms via binding to receptors with stereochemical specificity. Such pili have been found to adhere to abiotic surfaces, urinary catheters, bladder urothelium, and/or kidney tissue (*3*, *6*–*8*). Therefore, these CUP pili represent key virulence or colonization factors in pathogenesis.

CUP pilus two domain adhesins are made up of a pilin domain and a receptor binding domain (RBD) (*8*–*10*), which are β strand rich (*6*, *9*). The RBDs dictate stereochemical specificity of binding (*6*, *7*, *11*), and as such, studies of RBD structural biology have been shown to be important for the development of antibiotic sparing therapeutics (*8*, *11*). Characterization of binding pockets and the rational design of small molecule inhibitors leverages the structural features of adherence for the development of targeted small molecule and monoclonal therapies against adhesive factors, which can disrupt pathogen binding and colonization. For example, the characterization of FimH, the CUP adhesin critical for binding to bladder tissue and causing cystitis, has allowed the rational development of high-affinity receptor analogs, called mannosides (*11*), which are now undergoing clinical trials.

In addition to being found in the urinary tract of those suffering acute or recurrent UTIs (rUTIs), UPEC is found within the GIT (*3*, *4*), serving as a reservoir for uropathogens, seeding the perineum and eventually causing a UTI (*3*). It is also suggested that outbreaks of UTIs within a community may have food-borne origins (*12*). As such, the gut-bladder axis in UTIs is an area of great scientific interest. We recently showed that YehD_RBD_ can bind the luminal contents of colonic sections from mice (*13*). A recent work also indicates that *yeh* (Yeh pilus operon) expression is up-regulated in the GIT of patients with inflammatory bowel disease, suggesting a role of Yeh in *E. coli* colonizing a dysbiotic GIT (*14*). Correspondingly, patients with rUTI have been found to similarly have a dysbiotic gut (*4*).

The operons for Yeh and its sister Yeh-like (Yhl) pili are widespread throughout *E. coli* phylogroups. Although there is some evidence of Yeh’s role in the GIT, there has yet to be a detailed investigation of Yhl function. Furthermore, little is known about the Yeh and Yhl pilus structure or function specifically. In addition, although intestinal bacteria have been found to bind preferentially to particular food-derived carbohydrates within the intestine (*15*) and outbreaks of UTIs within a community are hypothesized to have food-borne origins (*12*), molecular details regarding how UPEC may bind to food-derived carbohydrates have yet to be greatly studied.

Here, we shed light on the structural and functional role of the Yeh CUP pilus in the GIT. We present the YehD_RBD_ and YhlD_RBD_ crystal structure with an RBD structural motif containing a relatively large, α-helical flap hinged at the distal tip of the RBD core. In using YehD, the more prevalent and widely shared adhesin in *E. coli*, as a model protein for studying this structural motif, we demonstrate that Yeh adheres to pectin, a component of foodstuffs contained within the colon, whereas YhlD binds to a receptor on the luminal surface of the colon. In silico and in vitro assays suggest that this flap can adopt open and closed conformations, although it likely favors the closed conformation in solution. Further modulation of the conformation ultimately influences the binding affinity of the protein itself. Last, our work demonstrates that YehD contributes to GIT colonization. This work provides insights into GIT colonization and reservoir formation by *E. coli* and highlights a possible mechanism by which UPEC can be introduced into a person’s gut through consumption of plant fibers to which UPEC is bound via Yeh pili.

## RESULTS

### Yeh and Yhl pili are related pili encoded by distinct populations of *E. coli*

**Fig. 1.**
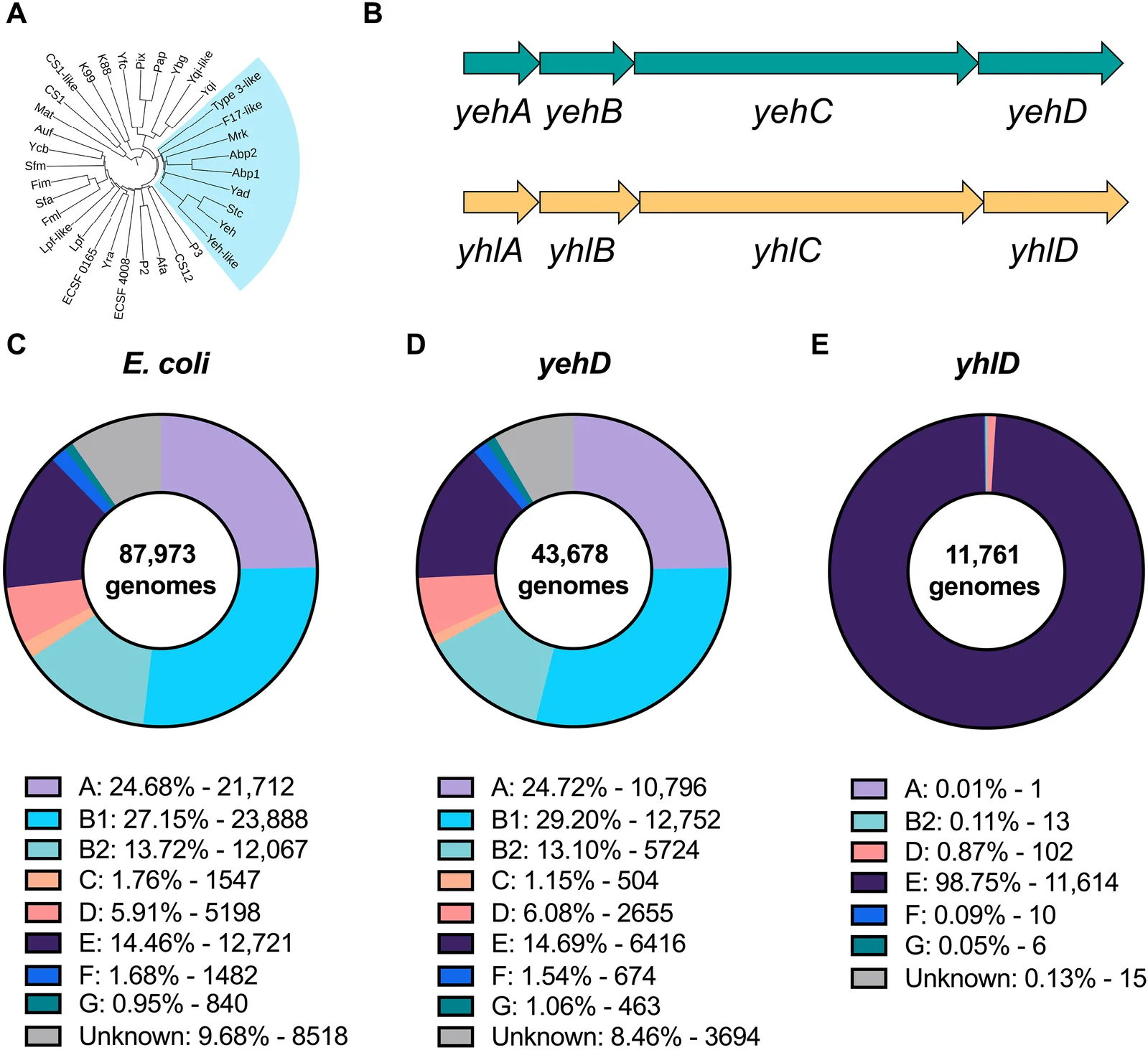
*Yeh* and *Yhl* operons are closely related. (**A**) Phylogeny of CUP pilus ushers, modeled from (*20*). The γ4 clade is highlighted in blue. (**B**) Representative operon organization and naming of *yeh* and *yhl*. Major subunit, *A*; chaperone, *B*; usher, *C*; adhesin, *D*. (**C**) Publicly available *E. coli* genomes, organized into *E. coli* phylogroups. (**D**) Prevalence of *yehD* among the *E. coli* phylogroups. (**E**) Prevalence of *yhlD* among the *E. coli* phylogroups.

Yeh and Yhl pili are two closely related pili, each falling within the γ4 clade of classical CUP pili based on usher primary amino acid sequence (Fig. 1A). They are also closely related to the *Salmonella* Stc pilus, known to contribute to GIT colonization (*16*) (Fig. 1A). The operons encoding these pili are notable for containing the minimum number of genes to produce a functional pilus with a tip adhesin: (i) the major subunit (*yehA*/*yhlA*), (ii) a periplasmic chaperone (*yehB*/*yhlB*), (iii) an outer membrane usher (*yehC*/*yhlC*), and (iv) the tip adhesin (*yehD*/*yhlD*), as shown in Fig. 1B.

We assessed the sequences of ~90,000 *E. coli* genomes, distributed across a number of *E. coli* phylogroups, with most being present in four phylogroups (B1, A, E, and B2) in descending order of prevalence (Fig. 1C). Phylogroup B2 is associated with UTI isolates as well as those able to colonize the GIT (*17*, *18*), and phylogroup E is understood to be predominantly intestinal as well (*19*). Within *E. coli*, *yehD*, the gene encoding the adhesin, is well represented, encoded in just under one-half (43,678/87,973) of all unique *E. coli* genomes (Fig. 1D), although this is lower than the estimated >90% in a limited study seen previously (*20*). In addition, this gene appears proportionally distributed across phylogroups in this analysis. Approximately 15% of all *yehD* genes are also present in the intestinal E phylogroup (*19*) (Fig. 1D). In contrast, *yhlD* is encoded in approximately one-eighth (11,761/87,973) of the unique *E. coli* genomes, similar to a previous report (*20*), while being almost exclusively encoded within the E phylogroup of *E. coli* (Fig. 1E). Correspondingly, >90% of phylogroup E *E. coli* (11,614/12,721) encode *yhlD*. Approximately one-half of the phylogroup E strains contain both *yehD* and *yhlD*, possibly suggesting that their translated proteins have distinct functions.

### YehD_RBD_ and YhlD_RBD_ crystal structure contains an α-helical flap motif

**Fig. 2.**
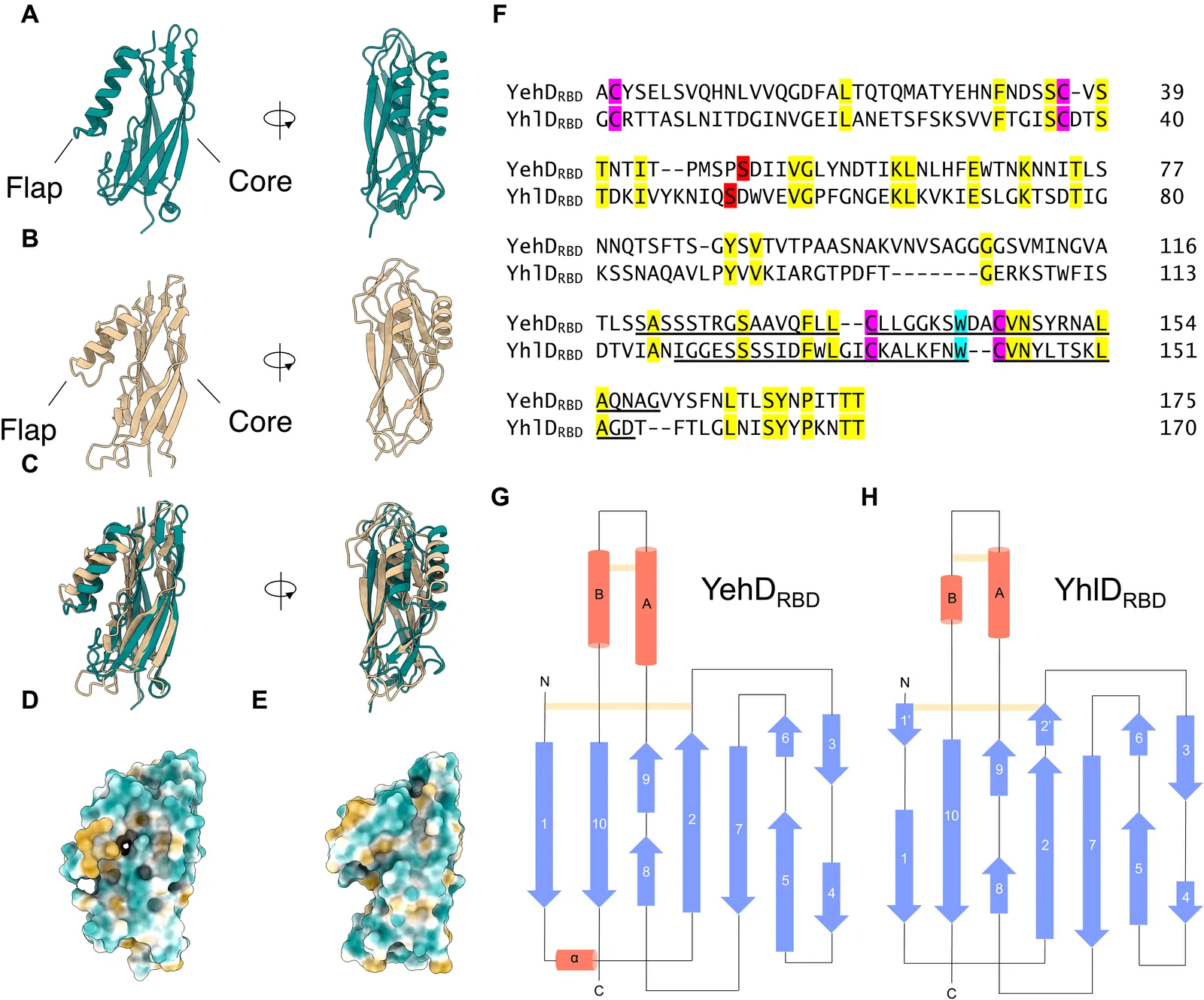
Crystal structures of YehD_RBD_ and YhlD_RBD_. (**A**) Cartoon model of YehD_RBD_ (Chain A), rotated 90° from left to right. (**B**) Cartoon model of YhlD_RBD_, rotated 90° from left to right. Core and flap motifs are labeled. (**C**) Overlaid cartoon models of YehD_RBD_ (turquoise) and YhlD_RBD_ (gold), rotated 90° from left to right. (**D** and **E**) Surface hydrophobicity models of YehD_RBD_ and YhlD_RBD_. Turquoise represents hydrophilic; gold represents hydrophobic. (**F**) Clustal V alignment of the primary structure of YehD_RBD_ and YhlD_RBD_. Cysteines contributing to disulfide bonds are represented in purple. Consensus residues are represented in yellow. Conserved serine at the lip of the candidate binding pocket is represented in red. Conserved tryptophan contributing to protein dynamics is represented in cyan. Flap and hinge residues are underlined. (**G** and **H**) Protein topology plots of YehD_RBD_ and YhlD_RBD_, respectively. β Strands are represented as blue arrows and numbered; α helices are represented by red cylinders and labeled with letters. Disulfide bonds are represented by tan bars. Secondary structural elements are defined by PyMol.

To investigate their structure, we cloned, expressed, and crystallized the N-terminal RBD structures of YehD and YhlD (YehD_RBD_ and YhlD_RBD_, respectively). The solved structures of a 1.7-Å YehD_RBD_ [Protein Data Bank (PDB) ID: 9N4G] in the *P*2_1_2_1_2 space group and a 2.29-Å YhlD_RBD_ (PDB ID: 8V9V) structure in the *I*2_1_2_1_2_1_ space group (table S1) showed that these RBDs have almost identical tertiary structures, in contrast to their primary structure, but greatly differ from other known RBD structures (Fig. 2, A to E). The remarkable structural similarity between these proteins is notable because of the level of divergence observed in the primary amino acid sequences, which are only 14.1% identical by Clustal V alignment (Fig. 2F). Thus, although these two proteins are structurally similar and closely related by usher, they exhibit a relatively high dissimilarity in primary structure in the adhesin RBD specifically. These features, in accordance with the genomic analysis, suggest the presence of divergent selective pressures and potentially different roles or functions for these RBD proteins.

Furthermore, YehD_RBD_ and YhlD_RBD_ are nearly identical with regard to their secondary structure, each being composed of 10 β strands or stretches of β strands (Fig. 2, G and H) folded into a common β strand “jelly roll” motif structure seen in other RBDs (*9*) (Fig. 2, A to C). However, they also contain a flap composed of two antiparallel α helices located between the β strands 9 and 10 in both structures and are oriented to open proximally toward the base of the pilus (Fig. 2, A to C, G, and H). Furthermore, the distal ends of these flaps are composed of hydrophobic residues, which is in contrast to the hydrophilic residues expected to coat the surface of a soluble protein (Fig. 2, D and E). We refer to this overall structural feature as the “flap motif” as it runs along the length of the protein core, covering a potential pocket (fig. S1, A and B). The lip of this pocket is located between β strands 3 and 4 and on which serine-49 (S49) (highlighted in red) sits (Fig. 2F). Y150 contributes to the posterior portion of this pocket as well, being situated on the second α helix on the flap motif. Holding together these secondary elements are two disulfide bonds, one near the N terminus with a second holding the α helices together in this CUP adhesin motif (Fig. 2, F to H). Although α-helical elements have been seen in other RBDs (*10*, *21*), this sort of α-helical motif has not been described previously in CUP adhesin RBDs.

Compared to canonical β strand–rich RBDs, the circular dichroism (CD) spectrum of YehD_RBD_ supports a structure with both α-helical and β strand elements. Between 200 and 220 nm, the spectra of the YehD_RBD_ contained a characteristic β strand peak. In the 220- to 240-nm range, the YehD_RBD_ spectrum remains depressed, indicative of α-helical elements. In combination, the 200- to 220-nm and 220- to 240-nm ranges for YehD_RBD_ are consistent with a spectrum exhibiting characteristic α-helical and β strand elements overlapping with each other (fig. S1C) and are consistent with the crystallographic model as described.

### YehD_RBD_ ensemble contains conformational heterogeneity

The respective flap motifs are connected to a β strand–rich core by a short distally oriented (from the pilin domain) hinge (Fig. 2, A to C). These hinges are rich in short R-group amino acids and do not contain prolines (Fig. 2F) and thus have the potential for flexibility. In both crystal structures, the flap motif runs along the long axis of the RBD core at a length of ~30 Å for YehD_RBD_ and YhlD_RBD_ compared to the ~50- to 55-Å-long cores (Fig. 2, A to C).

To assess the conformational heterogeneity of the α-helical flap, we performed all-atom molecular dynamics simulations on a preliminary model of wild-type (WT) YehD_RBD_. Specifically, we used the adaptive sampling scheme, FAST (fluctuation amplification of specific traits), on an intermediate model of YehD_RBD_ (fig. S1D) [root mean square deviation (RMSD) of 0.614 Å between 9N4G and State00] to sample conformations that maximized the distance between the α-helical flap and the RBD core. A Markov state model (MSM) was built from the final dataset, which clusters conformations into discrete states and details their equilibrium populations.

**Fig. 3.**
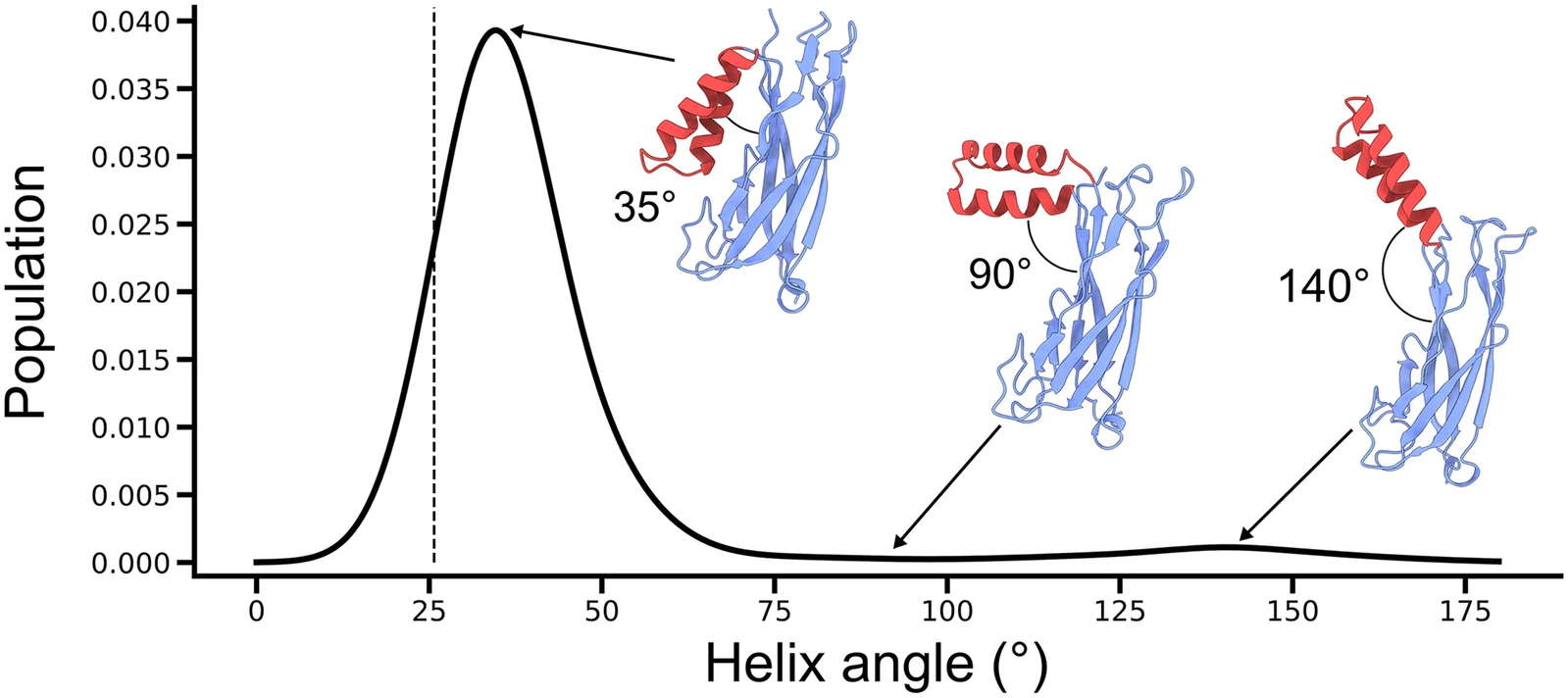
Conformational ensembles of YehD_RBD_ as predicted from FAST molecular dynamics simulations. Population of the MSM is projected onto the angle formed between the α-helical flap and the β strand domain. Representative structures at 35°, 90°, and 140° are depicted as cartoon, with the α-helical flap and β strand domain colored red and blue, respectively. The starting value of the angle from the initial structure is shown with a black dashed line.

Analysis of the MSM conformational landscape reveals that the α-helical flap motif has the potential for structural heterogeneity, in the form of “open” and “closed” states. The MSM projected onto the angle between β strand and α-helical domains shows that the flap motif can flip upward, with an angle of up to 180° with respect to the β strand core (Fig. 3). Although this motion is accessible, our MSM predicts open states to be at very low populations, with traversals to beyond the closed state being rare events. Furthermore, the MSM predicts that YehD_RBD_ spends 89.5% of its time with an opening angle less than 50°, with the initial input crystal structure having an angle of 25.7°. Thus, although the movement was possible, it was energetically unfavorable, and the closed flap orientation, as observed in the crystal structure, was favored under the conditions tested (Fig. 3).

**Fig. 4.**
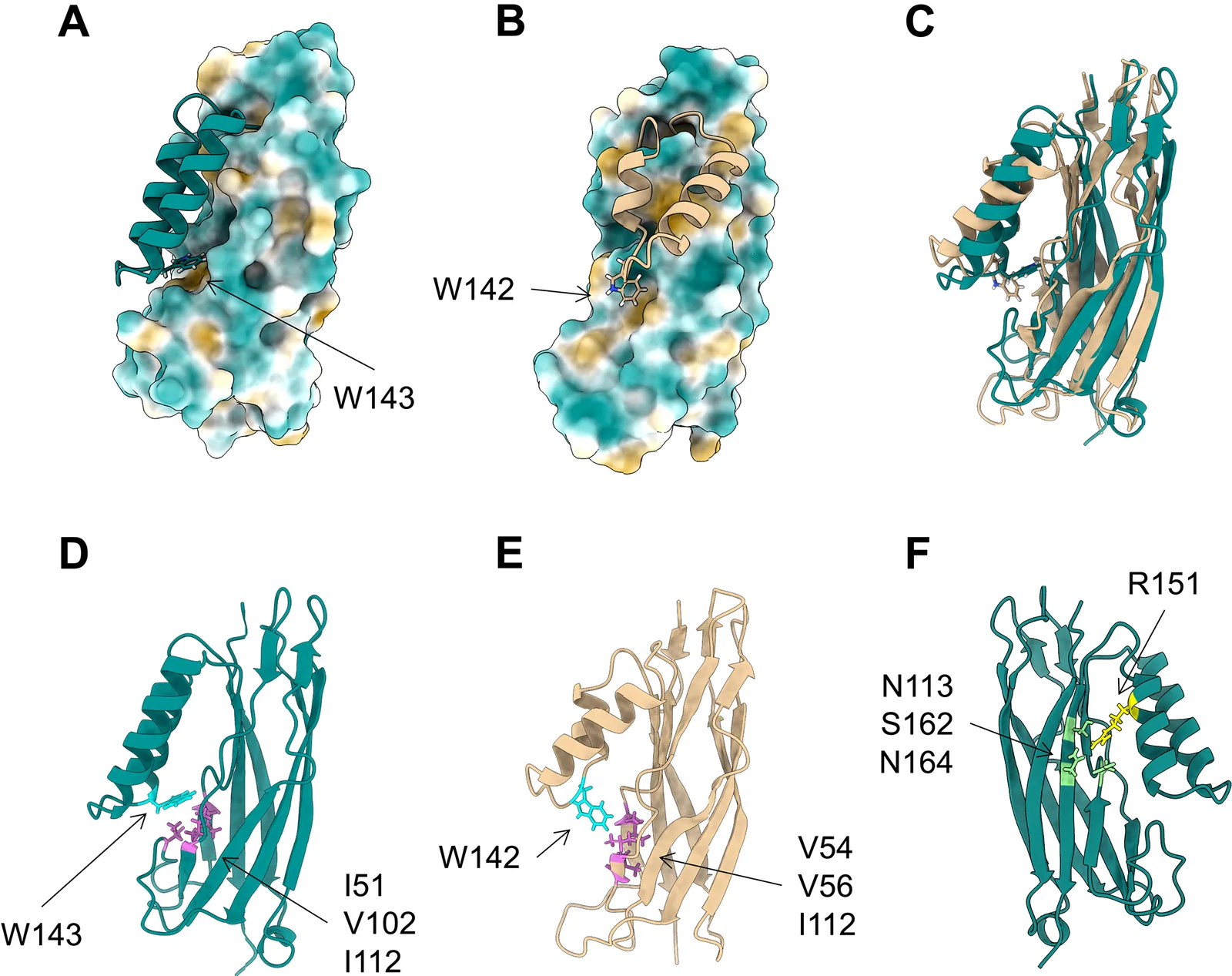
Residues of interest at the interface of the core and flap motifs. (**A** and **B**) YehD_RBD_ and YhlD_RBD_ with W143 and W142 highlighted, respectively. (**C**) Alignment of YehD_RBD_ (turquoise) and YhlD_RBD_ (gold) with W143 and W142 side chains shown. (**D**) YehD_RBD_ with W143 shown in cyan, which sits on the hydrophobic side chains of I51, V102, and I112 shown in purple. (**E**) YhlD_RBD_ with W142 shown in cyan, which sits on the hydrophobic side chains of V54, V56, and I112 shown in purple. (**F**) YehD_RBD_ (Chain B) with R151 oriented toward the pocket bound by N113, S162, and N164 (light green).

**Fig. 5.**
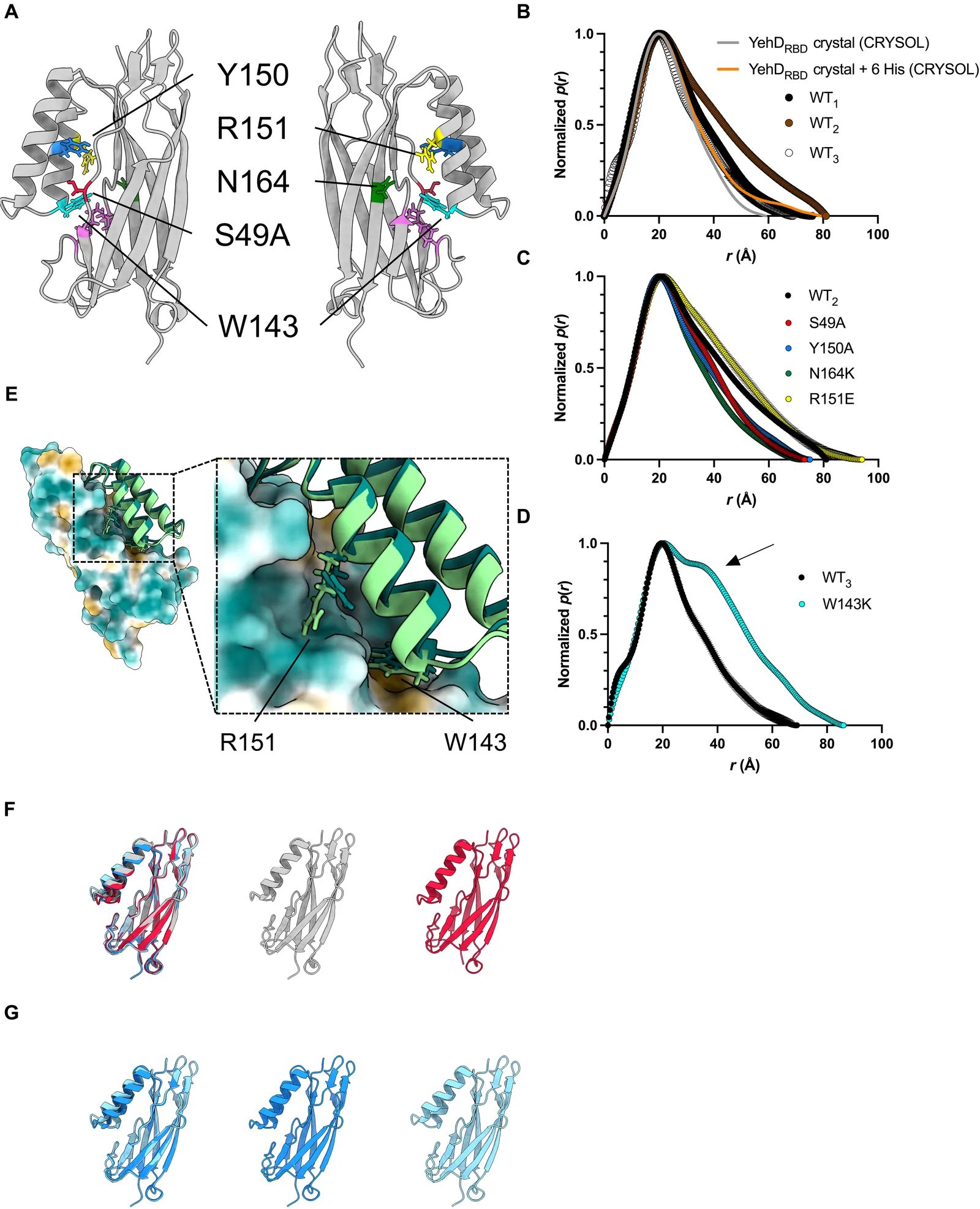
In solution structure of YehD_RBD_. (**A**) YehD_RBD_ (Chain A) rotated 180° from left to right with specific residues at the core-flap interface identified. In purple are I51, V102, and I112 of the hydrophobic patch. (**B**) Pairwise function [*p*(*r*)] function of coordinate models generated via CRYSOL and GNOM represented with lines. Three replicates of YehD_RBD_-6 His are represented by symbols. (**C**) *p*(*r*) of site-directed mutants of YehD_RBD_-6 His compared to WT control. (**D**) *p*(*r*) of site-directed mutant (W143K) of YehD_RBD_-6 His compared to WT. Reproducible notching is noted by an arrow. (**E**) YehD_RBD_ core from Chain A represented with the hydrophobic surface with flap motifs of Chain A (teal) and Chain B (light green) shown. (**F**) Left: Overlaid crystal structures, represented as a cartoon model, of YehD_RBD_ WT, S49A, and Y150A (Chains A and B). Middle: YehD_RBD_ WT (Chain A). Right: S49A (Chain A). (**G**) Left: Overlaid crystal structures, represented as a cartoon model, of YehD_RBD_ Y150A showing the flexibility of the flap. Middle: YehD_RBD_ Y150A (Chain A). Right: YehD_RBD_ Y150A (Chain B).

In examining the interface between the flap motif and the core, two particular regions were hypothesized to stabilize the closed conformation: (i) the “hydrophobic patch” generated by W143 on the end of the flap motif and I51/V102/I112 on the core (Fig. 4, A, C, and D) and (ii) the “hydrophilic insertion” of positively charged R151 on the flap motif and between three polar side chains (N113, S162, and N164) on the core midway up the flap motif (Fig. 4F). The tryptophan at the distal end of the flap is conserved between YehD (W143) and YhlD (W142) (Fig. 4, A to C), suggesting a conserved and critical function. To test the role of the hydrophobic patch and the conserved W143 in flap mobility, we made a W143K YehD_RBD_ mutant. To test the role of the hydrophilic patch in flap mobility, we made R151E and N164K YehD_RBD_ individual mutants (Fig. 5A). We hypothesized that the binding pocket region lies in a region between the flap and core of the RBD, creating YehD_RBD_ S49A and Y150A individual mutants to test this hypothesis. They also contributed as controls to the structural mutagenesis work as they represented relatively neutral mutations to alanine as compared to the more drastic biochemical changes attempted at other residues positions. All mutants, except for the YehD_RBD_ W143K variant, which had very low expression, expressed at levels similar to the WT YehD_RBD_ protein. Although crystallization trials were attempted for mutant proteins, only the S49A and Y150A variants produced crystals suitable for x-ray diffraction analysis.

Small-angle x-ray scattering (SAXS) analysis found that the suite of YehD_RBD_ mutants tested were appropriately folded compared to WT (fig. S2, A to C). However, analysis of the WT construct for de novo structure generation was variable and likely limited by the size of protein (~20 kDa) with this relatively low-resolution technique and a flexible histidine tag, which could be misinterpreted for flap movement (fig. S3). Thus, our SAXS analysis was limited to broader characterization of protein shape.

In contrast to WT, the protein variant W143K reproducibly (Fig. 5, B to D, and fig. S3D) exhibits a notched *p*(*r*) curve compared to WT and thus revealed a shape change consistent with an open conformation of the flap motif when comparing it to the in silico model states 5 to 10 (Fig. 5D and fig. S4), which represent open conformation protein states. The tryptophan-to-lysine mutation represents a switch from a hydrophobicity to hydrophilicity and thus likely caused a repulsive interaction with the hydrophobic patch resulting in the opening of the flap motif. These results suggest that the undisturbed hydrophobic patch contributes to the closed conformation.

N164K of the hydrophilic insertion region also appeared to largely mirror the WT control (Fig. 5C), although its reconstruction did show a flap-like mass extended at an ~90° angle (fig. S3). In addition, although R151E did exhibit a larger *D*_max_ compared to the other mutants assayed, its *p*(*r*) function shape is broadly consistent with WT and the change is *D*_max_ is reasonably explained by low resolution and imprecision of the technique at this protein size (Fig. 5C). However, much like N164K, a de novo reconstruction of the R151E data showed a flap-like mass extended from the protein core (fig. S3). As shown in Fig. 5E and fig. S5, within molecule A of the YehD_RBD_ crystal structure, the R151 side chain exhibited multiple orientations in the asymmetric unit at ~50% occupancy each. Nonetheless, the flap motif was closed. This demonstrates that R151 side-chain insertion into the protein core is not necessary for flap closure.

The *p*(*r*) function of WT construct of YehD_RBD_ was mirrored by S49A and Y150A (Fig. 5C). Thus, these mutations did not affect the conformation of the flap motif, consistent with their de novo reconstructions (fig. S3). Furthermore, the structures of YehD_RBD_ S49A and Y150A were solved to atomic resolution via x-ray crystallography (1.65 and 1.68 Å, respectively) (Fig. 5, F and G). When aligned with WT YehD (Chain A), these mutant proteins exhibited low RMSD values of 0.520 and 0.422 Å (Chain A for each), respectively. The diffraction data, across a number of mutants, under different crystallography conditions and two separate scattering techniques, suggest that a closed conformation is favored by the protein. This model is also consistent with the presence of a tryptophan molecule on the distal end of the flap, which likely prefers to be buried into the protein rather than solvent exposed.

### YehD_RBD_ binds pectin, influenced by the flap-core interface

**Fig. 6.**
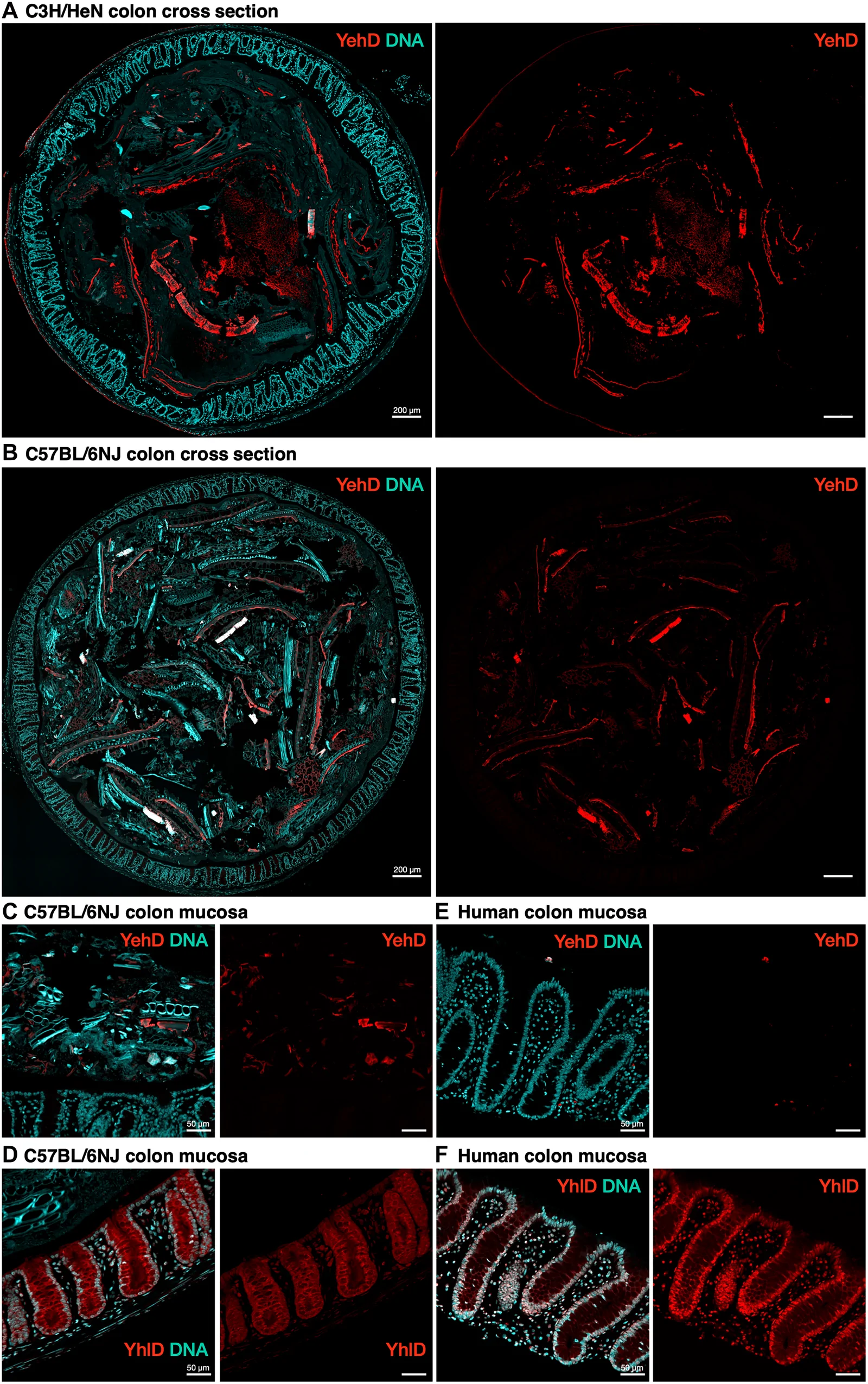
YehD_RBD_ and YhlD_RBD_ in situ binding in the GIT. Red: Alexa Fluor 657 WT protein conjugated to RBD. Turquoise: Hoechst staining. Shown on the left are overlaid images, and on the right is protein only staining. YehD_RBD_ WT staining to the colonic contents of (**A**) female C3H/HeN mice and (**B**) C57BL/6NJ mice, whole tiled colon shown. (**C** and **D**) C57BL/6NJ mice with YehD_RBD_ and YhlD_RBD_ staining, capture of mucosa and lumen. (**E** and **F**) Human colon sections with YehD_RBD_ and YhlD_RBD_ staining, capture of mucosa.

To investigate YehD and YhlD function, we performed staining with fluorescently labeled lectin domain on sectioned C3H/HeN and C57BL/6NJ mouse colons with intact colonic contents. Although we have previously shown that YehD_RBD_ can bind luminal contents (*13*), here we demonstrate that this phenotype holds with both C3H/HeN (Envigo) (Fig. 6A) and Taconic Lab C57BL/6NJ mice (Fig. 6, B and C), with staining exclusively located in the lumen of the GIT. Conversely, YhlD_RBD_ adheres throughout the mucosa, including to the epithelial surface and to cells within the crypts and laminal propria of murine sections, but does not bind to luminal contents (Fig. 6D). Although these samples do not include the colonic mucus or lumen contents, these results are recapitulated in human tissue sections, with YehD_RBD_ not staining the mucosa whereas YhlD_RBD_ does (Fig. 6, E and F), suggesting that its phenotype is consistent between species. When examining the colonic lumen, YehD_RBD_ binds to rigid, fibrous material, likely fibers derived from foodstuffs.

**Fig. 7.**
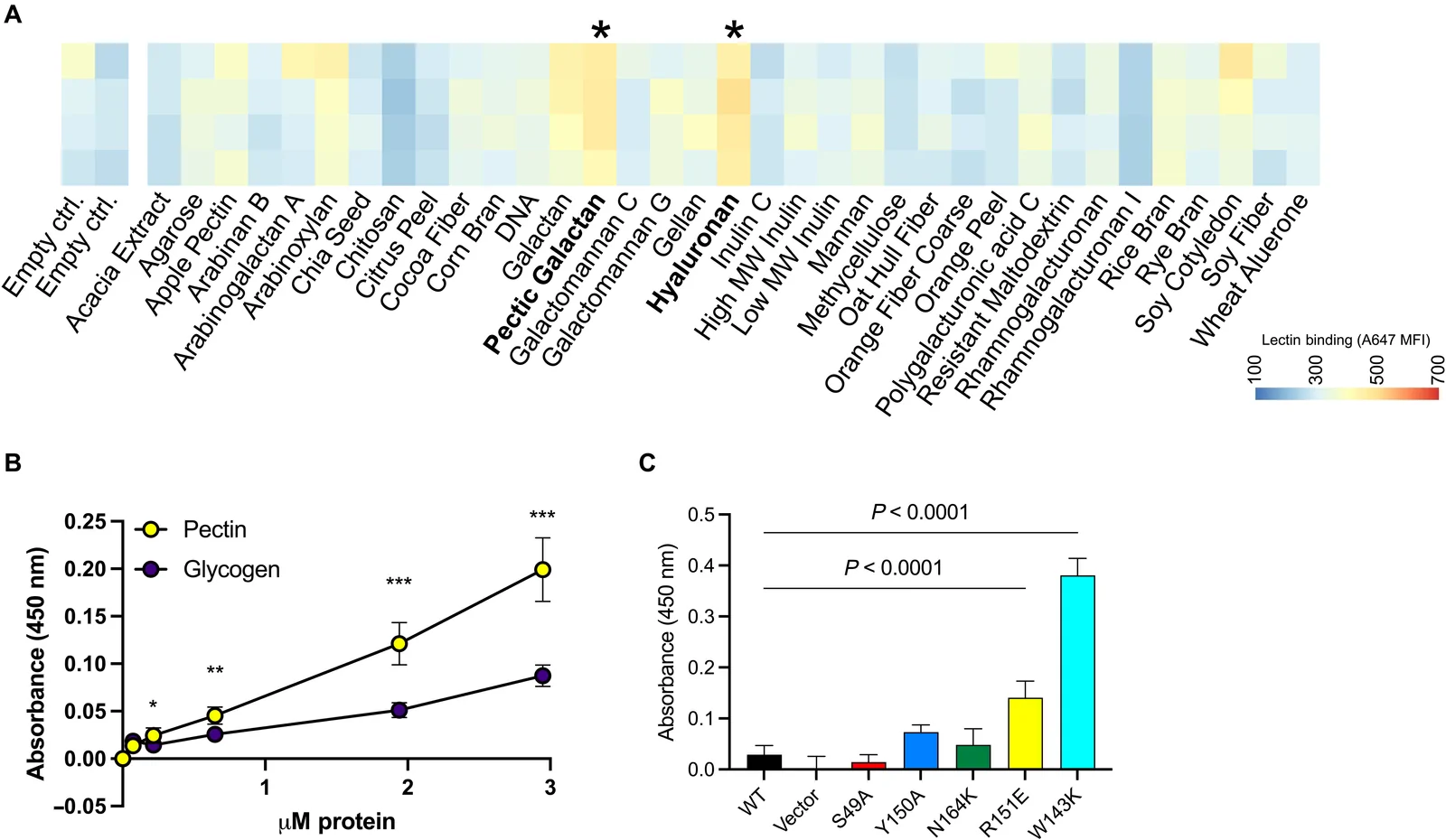
In vitro YehD_RBD_ binding. (**A**) Quantification of Alexa Fluor 647–labeled YehD_RBD_ binding to glycan-coated beads analyzed by flow cytometry. The geometric mean fluorescence intensity (MFI) in the Alexa Fluor 647 channel for each population of beads is indicated by the color bar. Columns denote the carbohydrate preparation coated on each bead type. Four replicate assays are shown in four rows. Background binding to empty beads was subtracted from the signal for each glycan bead type. Significant differences in binding between each glycan bead versus empty beads are indicated. *P < 0.05, *t* test, Bonferroni corrected. MW, molecular weight. (**B**) Biotinylated YehD_RBD_ adherence to two polysaccharides: pectin and glycogen. Pooled data from three independent experiments containing two replicates each. Statistical analysis with *t* tests with Welch’s correction shown. *P < 0.05; **P < 0.005; ***P < 0.0005. (**C**) Cells expressing Yeh tipped with WT and mutant YehD adherence to pectin. The representative replicate is shown. Statistical analysis with analysis of variance (ANOVA) with Brown-Forsythe and Welch tests and Dunnett’s T3 multiple comparisons test. *P* values are shown.

Mice are fed a chow composed of cereal based ingredients including corn, oatmeal, and soybean (*22*). To assess the binding of YehD to common classes of glycans in dietary plants, we screened a library of artificial food particles consisting of microscopic silica beads coated with preparations of biotinylated plant carbohydrates (*15*). We detected statistically significant binding of YehD_RBD_ to pectic galactan (composed of linear β-1,4-galactan attached to a pectic fragment containing rhamnose and galacturonic acid) (Fig. 7A and fig. S7). Pectin is a common ingredient in the plant cell wall, which provides structural rigidity to the cell (*23*). We also included several mammalian glycans in the bead library and observed binding to hyaluronic acid, which carries repeating negative charges, similar to the rhamnogalacturonan in pectin. Correspondingly, YehD_RBD_ bound with higher affinity to immobilized pectin as compared to glycogen in vitro (Fig. 7B).

We next assessed the mechanism of YehD receptor binding. We developed a cell-based assay with cells expressing Yeh pili from a plasmid. Site-directed mutations to *yehD* were made on the plasmids resulting in bacteria expressing Yeh pili with mutant adhesins. Expression of pili was comparable between the different assayed adhesin mutants (fig. S7). We initially hypothesized that S49 and Y150, two residues that contribute to an ~60-Å^3^ pocket located at the interface of the α-helical flap and the RBD core, each mutated to alanine, could interrupt binding and implicate this pocket as the interface for protein adherence. However, we did not observe a statistically significant defect in binding with these two mutants, leaving this region a candidate binding region pending further studies. Nonetheless, we found that expression of Yeh pili containing W143K or R151E YehD mutants displayed increased adherence compared to WT (Fig. 7C). These two mutants correspond to the hydrophobic patch and hydrophilic insertion interfaces between the core and flap motifs. As described above, the W143K mutant exists in an open conformation compared to WT, and as such, this result indicates that opening the flap increases affinity for the receptor. R151E increases affinity, although it does not appear to affect flap opening. Thus, R151 needs to be further assessed for its role in protein adherence. Overall, these data suggest that binding may be regulated by conditions that affect the opening of the flap to potentially allow access to a binding pocket.

### Yeh contributes to UTI89 GIT colonization in the absence of the type 1 pilus

**Fig. 8.**
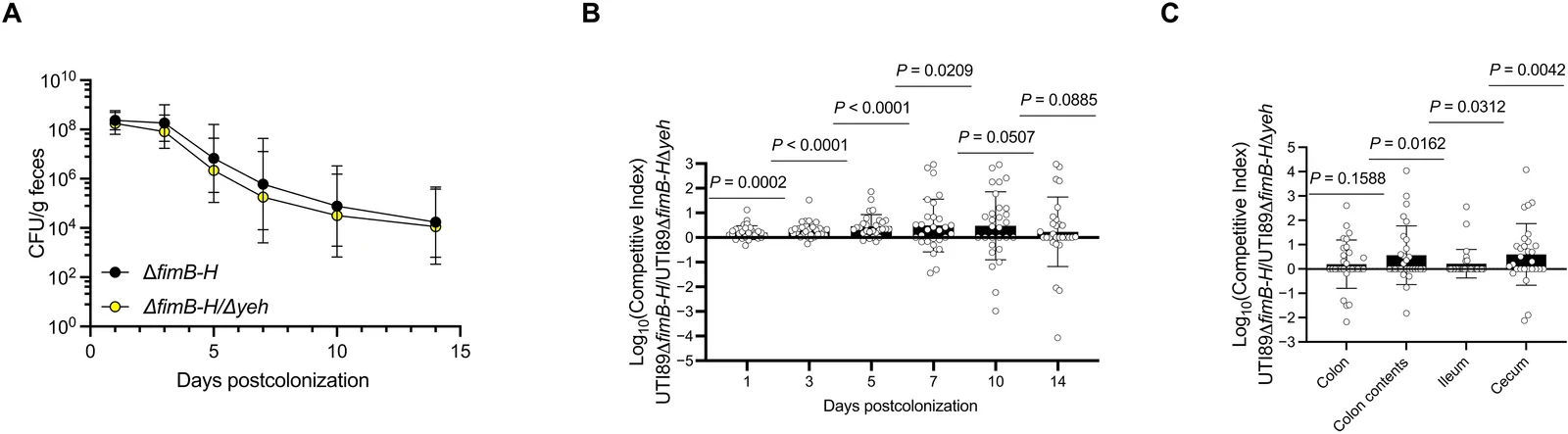
Yeh pilus contributes to GIT colonization in the absence of the type 1 pilus. (**A**) Individual titers of the competitive colonization experiment competing UTI89Δ*fimB-H* versus UTI89Δ*fimB-H*Δ*yeh*. *N* = 29 C3H/HeN mice over two separate experiments. (**B**) Log(Competitive Index) of UTI89Δ*fimB-H* versus UTI89Δ*fimB-H*Δ*yeh* for CFU/g feces in each mouse individually on each day of sampling. (**C**) Log(Competitive Index) of UTI89Δ*fimB-H* versus UTI89Δ*fimB-H*Δ*yeh* for CFU/g organ within each mouse on day 14. The Wilcoxon test (including the method of Pratt) was used to determine statistical significance compared to a null hypothesis value of zero. *P* values are shown above each dataset.

As YehD_RBD_ adheres to pectin, present in the GIT, we considered its in vivo role in the GIT. A previous screen of CUP pili mutants in UTI89 did not implicate Yeh in gut colonization (*3*). However, one possible explanation for this is that other pili, which promote GIT colonization, could mask the effect of deleting *yeh*. Type 1 pili are GIT colonization factors, and *fim* mutants in UTI89 are attenuated in GIT colonization in vivo, albeit colonization is still detected (*3*). We thus generated a UTI89 strain lacking both *fim* and *yeh* (UTI89Δ*fimB-H*Δ*yeh*) and analyzed its ability to compete for GIT colonization with UTI89 only lacking *fim*. These two strains display similar growth in vitro in LB Broth (fig. S9). We collected fecal samples to assess the status of colonization over time and harvested GI organs and contents after euthanasia. Both were able to colonize the GIT in the same mice at 14 days postinoculation in C3H/HeN pretreated with streptomycin (*3*) (Fig. 8A). However, we found that UTI89Δ*fimB-H*Δ*yeh* is at a competitive disadvantage against UTI89Δ*fimB-H* (Fig. 8B). The competitive advantage of UTI89Δ*fimB-H* over UTI89Δ*fimB-H*Δ*yeh* is present almost immediately, starting at day 1 postcolonization. The log competitive index (CI) represents an ~1.6-fold (on day 1) to ~3.1-fold (on day 7) higher relative colonization for UTI89Δ*fimB-H* compared to UTI89Δ*fimB-H*Δ*yeh* across all mice. Likely, the insignificant differences identified on days 10 and 14 are due to loss of high titer colonization on those days. At 2 weeks postcolonization, the differences are most notable in the colon contents (~3.6-fold difference) and cecum of the mice (~3.9-fold difference) (Fig. 8C). We were able to similarly show a small, but significant, defect in colonization, via fecal titers, in C57BL/6NJ mice on 3, 5, and 10 days postcolonization, although not in any particular organ type (fig. S9). Thus, under these conditions, Yeh appears to have a contribution to GIT colonization.

## DISCUSSION

The YehD_RBD_ and YhlD_RBD_ adhesin domains are structurally and mechanistically distinct from other structures of γ4 adhesin structures. YehD_RBD_ and YhlD_RBD_ not only contain the canonical β strand jelly roll motif but also have α-helical flaps, which contribute a large portion of the secondary structure of the proteins. Whereas other β strand–rich RBDs contain α-helical elements, like UclD_RBD_ (*3*), none do so to the extent of YehD_RBD_ and YhlD_RBD_.

Beyond their static structures, there is a growing list of examples by which CUP adhesins can regulate their ability to bind to their receptor ligands, suggesting a widespread need for RBDs to have titratable binding affinities. Dynamism is already well characterized in FimH (*10*), a γ1 adhesin, showing that FimH can exist in low-affinity and high-affinity conformations, as dictated by the contrasting features of the low-affinity and high-affinity binding pockets. This mechanism is regulated by the allosteric interaction between the pilin and RBDs of FimH and sheer forces, described as a catch bond mechanism (*24*). Closer relatives of YehD, in the γ4 clade, Abp1D and Abp2D adhesins from *Acinetobacter baumannii* also exhibit evidence of conformational dynamism with modulation of an anterior loop of its binding pocket increasing its affinity for its receptor (*6*). This loop is analogous to a loop adjacent to the candidate binding pocket of YehD. In YehD, a multidisciplinary approach has revealed the α-helical flap’s potential to flip up and away from the RBC β strand core, thus affecting that possible binding pocket region.

Inspection of the crystal structure revealed two potential locations that could be contributing to this conformation and ultimately function, a hydrophobic patch at the distal end of the α-helical flap and a hydrophilic interaction midway along the flap. In YehD_RBD_ W143K, disruption of the hydrophobic patch caused a notching in the *p*(*r*) function, indicating a shift of protein mass away from the protein core, in the W143K species population. Correspondingly, the W143K exhibited a higher affinity for pectin, suggesting that opening the flap increases protein affinity. On the other hand, manipulation of the hydrophilic insertion region does not appear to affect the dynamics of the protein flap but does appear to increase the affinity of the protein, requiring further investigation as to the exact mechanism.

Also of note, the proximal end of the RBD core has a 15-Å-wide depression, likely representing an interface where the pilin domain can interact with the RBD. Further structural studies are needed to assess whether a catch bond mechanism, as seen in FimH, also occurs in YehD, possibly affecting the interaction between the core and flap motifs. Such work could lend further insight into the dynamism of the flap motif in a pilus tip-like state.

Last, the GIT is a critical interface in the symbiosis between humans and their microbiota (*25*). Interruption of the resident flora allows for pathogens, like *Clostridium difficile* and potential uropathogens, to colonize the GIT (*5*, *26*). UPEC is known to use type 1 and F17-like pili to colonize the GIT in a murine model (*3*). Yeh is known to be up-regulated in a dysbiotic GIT (*14*); however, it was not previously shown to contribute to the competitive fitness of the uropathogen UTI89. We found that the deletion of *yeh*, in a background of a type 1 deletion, showed a small defect in GIT colonization against a control type 1 deletion only strain, suggesting that, in this particular model, Yeh’s role may be limited compared to the type 1 and F17-like pili. However, there may be a more substantial role to be investigated in dysbiotic models, a state in which Yeh is now known to be up-regulated (*14*).

We have also demonstrated previously (*13*), and within, the ability of YehD_RBD_ to bind luminal contents of the colon. We show that this phenotype likely extends to humans with the inability to bind to colonic mucosa, in contrast to YhlD_RBD_. Furthermore, with Yeh’s ability to adhere to pectin, a bacterium could use this pilus for environmental persistence on plant material, allowing a bacterium to use foodstuffs to traffic into and through the GIT.

Further study is warranted toward assessing the role of YhlD in the GIT as we have demonstrated that it can bind to GIT epithelial tissue. Yhl is not present in UTI89, but >90% of *yhlD* is present phylogroup E strains. These strains are thought to be predominantly intestinal (*19*), and YhlD could contribute to this phenotype. Thus, understanding more about its role in host pathogen interactions may reveal more about the mechanisms of phylogroup E pathogenesis as a whole. Because of conserved residues (W143 and S49 in YehD corresponding to W142 and S51, respectively, in YhlD), we expect that YhlD has a similar mechanism of conformational dynamism and binding, with a candidate pocket also sitting between the flap and core motifs.

The work within further contributes to our knowledge of YehD and YhlD structure and function. In doing so, we have developed the following model of Yeh function. Yeh is able to adhere to pectin in plant material, either within or outside the GIT. It adheres via an RBD with an α-helical flap, which has not previously been described. This flap favors a closed conformation due to hydrophobic interactions between the core and flap. The YehD_RBD_ sits atop a Yeh pilus that allows for GIT colonization in the host. Overall, Yeh contributes to maintaining a uropathogen reservoir in the GIT, demonstrating that the Yeh pilus is among a growing number of recognized GIT colonization factors.

## MATERIALS AND METHODS

### Cell growth

Bacteria were grown at 37°C with shaking in LB broth with or without corresponding antibiotics. Stocks were stored at −80°C in glycerol.

### Growth curves

Strains were inoculated as single colonies from LB agar plates into LB media and grown overnight under shaking conditions at 37°C for ~12 to 16 hours. Cultures were harvested and diluted to 0.01 OD_600_ (optical density at 600 nm) in fresh LB. The bacteria were then loaded in a Corning 96-well flat-bottom plate, with 200 μl of diluted culture per well. The plate was read using a BioTek Synergy H1 plate reader at 37°C with shaking conditions over a period of 24 hours.

### Phylogenetic analysis

To interrogate for the presence of *yehD* and *yhlD*, a publicly available and searchable k-mer database constructed from 661K bacterial genomes (*27*) was queried using the COBS search index (*28*) (https://github.com/bingmann/cobs) with a threshold value of 0.90. Genomes were previously assembled and refined with Kraken 2 and Bracken to determine taxonomic species. *E. coli* phylotypes were previously determined using clermonTyping v1.4.1 (*29*). Genomes that were classified as “*E. coli*” but whose phylotype was inconsistent with this assignment (i.e., “Non*-Escherichia*”) were excluded from subsequent analysis. The phylogenetic tree was generated using the Interactive Tree of Life (itol.embl.de) (*30*). Primary structure alignment was performed using MegAlign.

### Cloning

The DNA sequence corresponding to the N-terminal 195 amino acids of the YehD adhesin was amplified from the *E. coli* UTI89 strain using paired primers containing a C-terminal 6x-his encoding sequence. This was subsequently inserted into the pTRC99a expression vector at the EcoR1 restriction site under control of the lac promoter using the InFusion protocol (Clontech Takara). The final cloned sequence was sequence verified to represent the lectin domain and signal sequence of the *E. coli* UTI89 YehD protein and was transformed into *E. coli* DL41 (BE3) methionine auxotrophic cells for expression. The his-tagged YhlD lectin domain for structural studies was generated by cloning the N-terminal 190 amino acids of the Sakai YhlD adhesin with a C-terminal 6x-his tag into pTRC99a using the InFusion protocol (Clontech Takara). Resulting plasmids were sequence verified and transformed into *E. coli* C600 cells. Site-directed mutagenesis studies were created using modified QuikChange methods. Briefly, a synthesized primer containing the nucleotide mutation and flanked by homologous regions to the *yehD* gene was used to amplify the entire plasmid by PCR (polymerase chain reaction). The resulting plasmid product was digested with DpnI to remove the template plasmid and transformed into DH5α cells. The presence of the intended mutation was validated by sequencing and then transformed into C600 cells for expression. Deletion of *fimB-H* from UTI89 WT and UTI89Δ*yeh* (*3*) was accomplished via linear recombination using λ red recombinase.

### Protein expression and purification

The WT and mutant YehD_LD_ constructs were expressed as indicated previously (*6*). Briefly, cells were grown to mid-to-late logarithmic growth phase in a medium of LB and Superbroth with ampicillin and induced with ~80 μM or more of isopropyl-β-d-thiogalactopyranoside (IPTG). Cells were induced at 37°C with shaking for 1 hour or overnight at room temperature with no shaking. The periplasm was harvested by digesting cells, resuspended in 20 mM Tris (pH 8) + 20% sucrose, with lysozyme and EDTA. The EDTA was removed via either salt precipitation or dialysis into 1x phosphate-buffered saline (PBS) + 250 mM NaCl. Proteins were purified by a cobalt affinity column, eluted with 1x PBS + 300 mM imidazole. An S column purification could be subsequently conducted for additional purification and concentrating. The S column was run with 20 mM MES (pH 5.7) and eluted with 20 mM MES (pH 5.7) + 500 mM NaCl. For expression of selenomethionine-containing YehD for experimental phasing, *E. coli* DL41(BE3) cells transformed with the 6xhis-YehD_RBD_ plasmid were grown in chemically defined M9PO4 media supplemented with amino acids and trace minerals and glycerol as previously described. Methionine was added in limiting amounts, and the culture was grown in a New Brunswick fermenter until growth plateaued at an OD of 2.5. At that time, selenomethionine (0.1 g/liter), 0.1 mM IPTG, and 1% (v/v) glycerol were added. After 1 hour of induction, the cells were harvested and the protein purified as described above. Native expression of YhlD_RBD_ was performed as described above using transformed C600 cells. FimH preparation and purification were conducted as described previously (*31*).

### Crystallographic studies

Selenomethionine-containing YehD_RBD_ [12.7 mg/ml in 20 mM MES (pH 5.8)] was crystallized in 0.2 M AmH_2_PO_4_ + 20% (w/v) polyethylene glycol, molecular weight 3350 (PEG-3350) via a hanging drop vapor diffusion method. Crystals were cryoprotected in a solution of mother liquor + 20% glycerol. The final crystal structure deposited was from a separate crystal from the preliminary model used for molecular dynamics modeling. YehD_RBD_ S49A and Y150A in 20 mM MES (pH 5.79) were crystallized in 0.2 M magnesium formate + 15% PEG-3350 at 4.37 mg/ml and 0.1 M sodium acetate, 0.1 M Tris (pH 8.8), and ~20% PEG-4000 at 7.18 mg/ml, respectively, via hanging drop. Harvested crystals were cryoprotected in ~16% glycerol + 0.313 M magnesium formate + ~16% PEG-3350 and 20% glycerol + 0.1 M Tris (pH 8.8) + ~15% PEG-4000 + 0.4 M sodium acetate for S49A and Y150A, respectively. YhlD_RBD_ in 10 mM MES (pH 5.8) at ~3 to 4 mg/ml was crystallized via a hanging drop method over 95 mM sodium citrate (pH 5.6), 5% glycerol, 19% PEG-4000, and 19% isopropanol. Harvested crystals were cryoprotected with mother liquor + 20% glycerol. Selenomethionine-containing YehD_RBD_ data were collected at the selenium peak at the ALS beamline 4.2.2. Native datasets were collected for YhlD_RBD_ and YehD_RBD_ S49A and Y150A. Data were processed using XDS. The YehD_RBD_ WT structure was phased by single-wavelength anomalous diffraction in Phenix’s Phaser program. S49A and Y150A were phased by molecular replacement using YehD_RBD_ WT. S49A diffraction data were indexed in *P*121 and solved in *P*12_1_1. YhlD_RBD_ was phased using an AlphaFold model of YhlD_RBD_ in Phenix. Data were subject to iterative rounds of refinement using Phenix and Coot. Data and collection statistics for these structures can be found in table S1. Diffraction and coordinate data were submitted to the PDB (PDB IDs: YehD_RBD_ WT, 9N4G; YehD_RBD_ S49A, 9N4H; YehD_RBD_ Y150A, 9N4I; and YhlD_RBD_ WT, 8V9V). Protein representations were aligned in PyMol and captured from ChimeraX. Secondary structure topography was created in Microsoft PowerPoint modeled on the “Pro-origami” server (*32*).

### Candidate pocket determination

Molecules of the crystal structures of YehD_RBD_ and YhlD_RBD_ with all waters and soluble ligands removed were submitted to CASTp 3.0 (Computed Atlas of Surface Topography of Proteins) with the radius probe set to 1.4 Å. Images were captured from the online user interface.

### FAST molecular dynamics analysis

Molecular dynamics simulations, run on a preliminary model of YehD_RBD_, were run with Gromacs 5.1.4 at 300 K using the AMBER-03 force field with explicit TIP3P solvent (*33*–*35*). The initial structure was placed in a dodecahedron box that extends 1.0 Å beyond the protein in any dimension. The entire system was then solvated and energy minimized with a steepest descents algorithm until the maximum force fell below 100 kJ/mol per nanometer using a step size of 0.01 nm and a cutoff distance of 1.2 nm for the neighbor list, Coulomb interactions, and van der Waals interactions. For production runs, all bonds were constrained with the LINCS algorithm and virtual sites were used to allow a 4-fs time step (*36*, *37*). Cutoffs of 1.0 nm were used for the neighbor list, Coulomb interactions, and van der Waals interactions. The Verlet cutoff scheme was used for the neighbor list. The stochastic velocity rescaling (*v*-rescale) (*38*) thermostat was used to hold the temperature at 300 K. Conformations were stored every 20 ps.

The FAST algorithm (*39*) was used to enhance conformational sampling of the α-helical flap motif. FAST-distance simulations were run for 75 rounds with 10 simulations per round, where each simulation was 80 ns in length (60 μs of aggregate simulation). The FAST-distance ranking function favored restarting simulations from states that maximized distances between residues of the α-helical flap (135 to 146) and their contacting residues on the β strand domain (49, 51, 101, 102, 112, 113, and 115). In addition, a similarity penalty was added to the ranking to promote conformational diversity in starting structures, as has been described previously (*40*).

MSMs were built from the FAST simulation data using enspara (*41*). An MSM is a network representation of a free-energy landscape, where nodes are discrete conformational states and directed edges are conditional transition probabilities. The state space was defined using backbone heavy atoms (atoms C, C_α_, C_β_, N, and O), which was clustered with a *k*-center algorithm based on the RMSD between conformations until every cluster center had a radius less than 2.2 Å. Centers were then refined using greedy *k*-medoids updates for five rounds. Following clustering, an MSM was built by row normalizing the observed transition counts, at a lag time of 1 ns, with a small pseudocount as a prior (*42*).

### In silico generation of *p*(*r*) curves

Crysol was used to generate theoretical scattering datasets for the intermediate states from the FAST molecular dynamics analysis. AutoRg in Raw was used to determine the radius of gyration, and Autognom in Raw was used to determine the pairwise function. Chimera was used to generate the 6-his model of the YehD_RBD_ crystal structure.

### SAXS analysis

WT and mutant protein constructs were dialyzed into 20 mM MES (pH 5.79) + 50 mM NaCl with or without ~1% glycerol. After dialysis, the proteins were concentrated with a goal of 5 mg/ml or greater. Using the high-throughput (HT) mail-in SAXS service at the Lawrence Berkeley National Laboratory, protein samples were sent in at three or four different concentrations with a buffer control for scattering subtraction. Data were processed using frameslice to account for radiation damage and merge data. Data were extrapolated to a zero concentration to account for protein concentration effects in Primus. The Guinier region was determined in RAW (2.1.4) and checked for general agreement with Primus AutoRg. Pairwise *p*(*r*) function was generated in RAW, assessing for *D*_max_ by unbounding the function from reaching zero and determining the *D*_max_ that the function most closely approached zero on a downward slope before becoming negative or beginning to rise again. In the event that the *p*(*r*) did not fit these criteria, the *D*_max_ was determined from the most favorable chi-square value of the fit of the function. Three-dimensional models were generated using DAMMIN in RAW. DAMAVER in RAW was used to average the envelope generated from DAMMIN.

### Protein biotinylation

YehD constructs were buffer exchanged or dialyzed into 1x PBS for biotinylation. Individual proteins were biotinylated with a ratio of 20:1 biotin reagent:protein. Biotinylation occurred at 4°C with agitation for 2 hours, and then the proteins were dialyzed into either 1x PBS or 20 mM MES (pH 5.79) + 50 mM NaCl.

### In situ staining

For mouse colon sections, paraffin-embedded, methanol-Carnoy fixed distal colon tissues from 7- to 8-week-old female C57BL/6NJ (Taconic Biosciences) and 7- to 8-week-old female C3H/HeN (Envigo) mice were sectioned to 5 μm and used for staining as described previously (*43*). Human colon sections were generated from healthy donors, ranging from age 56 to 70, and processed as previously described (*44*). This research was approved by the University of Gothenburg branch of the Swedish Ethical Review Authority (136-12). Participants provided informed consent, and samples were obtained at the Sahlgrenska University Hospital in Gothenburg, Sweden (*45*). Mouse and human colon sections were stained with Hoechst (5 μg/ml) with and without Alexa Fluor 647–labeled WT YehD_RBD_ or YhlD_RBD_ (10 μg/ml) in PBS. A 20X air objective lens was used to capture serial *z*-stacks spanning the entire section, and a maximum intensity projection (*x*/*y*) of the stack was generated for each channel. C57BL/6NJ colon sections were captured in the Department of Medical Biochemistry and Cell Biology at the Institute of Biomedicine, University of Gothenburg in Gothenburg, Sweden, and imaged using a Zeiss LSM700 confocal microscope. C3H/HeN colon sections were captured at the Wash. U. Center for Cellular Imaging and imaged on a Nikon W1 SoRa spinning disk confocal microscope.

### Adhesin binding screen using glycan-coated beads

Glycan-coated beads were generated and analyzed as described previously (*15*, *46*). Briefly, glycan preparations (5 mg/ml) were biotinylated using TFPA-PEG3-biotin (Thermo Fisher Scientific). Biotinylated glycans were incubated with paramagnetic, ~15-μm-diameter streptavidin-coated silica beads (LSKMAGT, MilliporeSigma) for 1 hour at room temperature, followed by a second incubation with biotinylated glycans. Beads were subsequently incubated for 30 min at room temperature with streptavidin-fluorophore mixtures (5 μg/ml) in HNTB buffer [10 mM Hepes, 150 mM NaCl, 0.05% Tween 20, and 0.1% bovine serum albumin (BSA)]. Equal numbers of uniquely labeled beads were then pooled into a single mixture. Alexa Fluor 647–labeled adhesins were added into a 5-μl aliquot of the pooled bead library to a final concentration of 40 μg/ml, and the solution was mixed by repeated pipetting. The suspensions were incubated in the dark at 4°C with constant rotation for 30 min; beads were then collected using a magnetic rack and washed three times with 100 μl of HNTB. Beads were analyzed on an Attune NXT analyzer (Thermo Fisher Scientific), their glycan coating was identified on the basis of streptavidin fluorescence, and Alexa Fluor 647 fluorescence was measured for each bead type. Parallel samples of the glycan bead library were incubated with buffer only to establish background fluorescence.

### ELISA binding studies

For glycan studies, 50 μl of citrus pectin and glycogen (20 mg/ml) were adhered to their respective wells. These glycans were blocked with 2% BSA in 1x PBS with 0.05% Tween 20. For protein binding studies, YehD_RBD_ was incubated separately in this blocking buffer as well. Protein was adhered to pectin or glycan-coated wells overnight at 4°C. After washing, wells were subsequently probed with streptavidin–horseradish peroxidase (HRP) and developed with the associated 1:1 developing reagent kit (BD OptEIA TMB Substrate Reagent).

For bacterial binding to pectin, bacteria were grown to mid-log phase growth and then induced at room temperature overnight in LB supplemented with 100 μM IPTG. Pectin (10 μg/ml) was bound to an enzyme-linked immunosorbent assay (ELISA) plate overnight and blocked for 1 hour in 1x PBS + 1% BSA. Bacteria were normalized to OD_600_ = 1.0 and were allowed to bind to a blocked pectin plate for 1 hour, washed three times in 1x PBS with 0.05% Tween 20, and fixed with 10% formalin for 15 min. Adherence was quantified by rabbit anti–*E. coli* polyclonal sera (1:500), washed three times with 1x PBS with 0.05% Tween 20, followed by incubation with anti-rabbit-HRP (1:1000; Seracare), and washed three more times before development. To measure the surface exposure of the YehD adhesin, OD_600_ = 1.0 bacteria were adhered to a blank ELISA plate for 1 hour and fixed with 10% formalin for 10 min, and YehD was detected with rabbit anti-YehD_RBD_ lectin domain sera (1:1000) for 1 hour, washed three times, followed by anti-rabbit-HRP (1:1000; Seracare) for 1 hour, and another 3x wash. HRP detection for both assays was developed as above.

### In vivo GIT colonization experiments

GI colonization experiments were conducted as previous reported (*3*) and approved by the Washington University Institutional Animal Care and Use Committee (IACUC) (protocol numbers 21-034 and 24-0279; assurance number D16-00245). Seven- to 8-week-old female C3H/HeN mice were from Inotiv (formerly Envigo), and 7- to 8-week-old female C57BL/6NJ mice were from the Jackson Laboratory. They were treated with streptomycin 24 hours before colonization. Mice were orally gavaged ~1:1 with 10^8^ cells each of their respective mutants. Fecal colony-forming units (CFUs) were determined at the indicated days. At 14 days, mice were euthanized, and their organs were harvested. CFU titers were plotted as geometric mean with geometric SD. CIs were calculated by dividing the reference strain by the mutant strain and determining the log_10_ of that value. Statistics were calculated on the log_10_(CI) values using a Wilcoxon signed-rank test against the null hypothesis of 0 as no difference in colonization would result in a CI of 1 and a log_10_1 would equal 0. The Pratt method was used to account for values which equaled 0.

### Circular dichroism

Assessed protein was dialyzed into 20 mM phosphate (pH 7.00). The dialysis buffer was saved as a buffer control for the CD assay. The assay was performed at a concentration of ~50 μg/ml. A 150-μl sample was placed into a cuvette (Starna Cell 16.100F-Q-10/Z15) and read for 7 s, with 1° steps, from 200 to 280 nm on a Chirascan spectrometer unit. Buffer subtraction was performed in GraphPad Prism.
