# Supplementary material for: The Yeh pilus adhesin is equipped with an α-helical flap motif, which contributes to pectin adherence
Source: Sci Adv. 2026 Jan 7;12(2):eadz1301. doi: 10.1126/sciadv.adz1301 (PMC12778044; doi:10.1126/sciadv.adz1301)
Supplement: Supplementary file 1 [file sciadv.adz1301_sm.pdf]

Science Advances
AAAS

# Supplementary Materials for

## The Yeh pilus adhesin is equipped with an α-helical flap motif, which contributes to pectin adherence

Kevin O. Tamadonfar *et al.*

Corresponding author: Scott J. Hultgren, hultgren@wustl.edu

*Sci. Adv.* **12**, eadz1301 (2026)
DOI: 10.1126/sciadv.adz1301

**This PDF file includes:**

Table S1
Figs. S1 to S9

## Table S1: Refinement Statistics of Crystallographic Data

| | YehD(RBD) WT (9N4G) | YehD(RBD) S49A (9N4H) | YehD(RBD) Y150A (9N4I) | YhlD(RBD) WT (8V9V) |
|---|---|---|---|---|
| Resolution range | 45.31 - 1.7 (1.761 - 1.7) | 46.15 - 1.65 (1.709 - 1.65) | 39.2 - 1.68 (1.74 - 1.68) | 40.82 - 2.29 (2.372 - 2.29) |
| Space group | P 21 21 2 | P 1 21 1 | P 1 21 1 | I 21 21 21 |
| Unit cell | 102.787 50.486 71.877 90 90 90 | 34.852 92.296 44.939 90 91.685 90 | 78.351 31.613 87.873 90 93.17 90 | 28.302 104.117 131.52 90 90 90 |
| Total reflections | 288449 (25904) | 125364 (12563) | 162681 (16626) | 18428 (1807) |
| Unique reflections | 41949 (4127) | 33872 (3066) | 46949 (4386) | 9217 (904) |
| Multiplicity | 6.9 (6.3) | 3.7 (3.7) | 3.5 (3.5) | 2.0 (2.0) |
| Completeness (%) | 98.83 (99.78) | 98.04 (90.71) | 92.86 (89.27) | 99.91 (100.00) |
| Mean I/sigma(I) | 15.66 (0.75) | 8.02 (0.66) | 8.85 (0.96) | 9.69 (1.03) |
| Wilson B-factor | 23 | 14.72 | 20.26 | 37.72 |
| R-merge | 0.1019 (2.169) | 0.1679 (2.143) | 0.1095 (1.426) | 0.06604 (0.687) |
| R-meas | 0.1102 (2.367) | 0.1967 (2.511) | 0.1298 (1.682) | 0.09339 (0.9716) |
| R-pim | 0.04171 (0.9386) | 0.1016 (1.296) | 0.06883 (0.8859) | 0.06604 (0.687) |
| CC1/2 | 0.999 (0.388) | 0.994 (0.297) | 0.995 (0.414) | 0.996 (0.515) |
| CC* | 1 (0.747) | 0.999 (0.677) | 0.999 (0.765) | 0.999 (0.825) |
| Reflections used in refinement | 41468 (4119) | 33451 (3066) | 46334 (4383) | 9214 (904) |
| Reflections used for R-free | 2028 (205) | 1598 (136) | 2338 (247) | 453 (41) |
| R-work | 0.2379 (0.3382) | 0.2047 (0.3221) | 0.2251 (0.3097) | 0.2192 (0.3234) |
| R-free | 0.2597 (0.3827) | 0.2391 (0.3579) | 0.2582 (0.3477) | 0.2622 (0.3904) |
| CC(work) | 0.954 (0.646) | 0.958 (0.642) | 0.939 (0.660) | 0.948 (0.675) |
| CC(free) | 0.937 (0.550) | 0.961 (0.507) | 0.918 (0.606) | 0.959 (0.672) |
| Number of non-hydrogen atoms | 2646 | 2638 | 2628 | 1262 |
| macromolecules | 2580 | 2536 | 2554 | 1245 |
| ligands | 12 | 28 | 12 | 0 |
| solvent | 54 | 74 | 62 | 17 |
| Protein residues | 346 | 345 | 348 | 167 |
| RMS(bonds) | 0.005 | 0.014 | 0.012 | 0.003 |
| RMS(angles) | 1.06 | 1.33 | 1.42 | 0.47 |
| Ramachandran favored (%) | 97.65 | 98.82 | 98.25 | 96.97 |
| Ramachandran allowed (%) | 2.35 | 1.18 | 1.75 | 3.03 |
| Ramachandran outliers (%) | 0 | 0 | 0 | 0 |
| Rotamer outliers (%) | 2.73 | 1.4 | 2.41 | 1.48 |
| Clashscore | 1.37 | 3.18 | 3.57 | 0.41 |
| Average B-factor | 28.98 | 18.88 | 26.94 | 41.75 |

| | | | | |
|---|---|---|---|---|
| macromolecules | 29.07 | 18.81 | 26.98 | 41.88 |
| ligands | 27.69 | 22.68 | 28.23 | |
| solvent | 25.03 | 19.73 | 25.38 | 32.08 |
| Number of TLS groups | 1 | 1 | 1 | 1 |

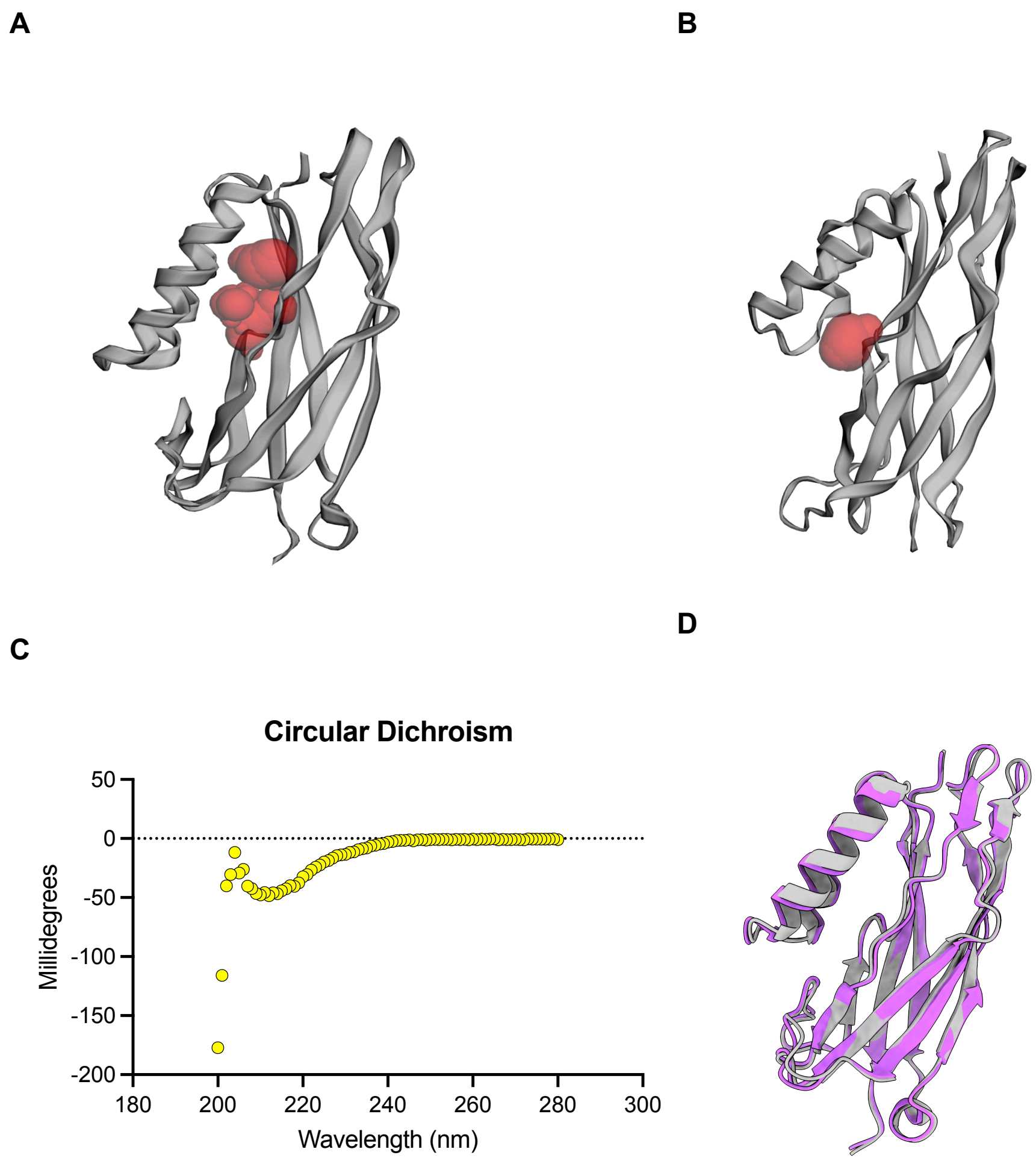


**Fig. S1: Structural features of $YehD_{RBD}$ and $YhlD_{RBD}$. A, B**) CASTp representations of $YehD_{RBD}$ and $YhlD_{RBD}$ respectively. Red represents possible pockets in each structure. **C**) Circular dichroism of $YehD_{RBD}$-6 His. **D**) Overlain $YehD_{RBD}$ crystal structure (PDB ID: 9N4G) (Gray) with State00 (Purple) generated from preliminary model of $YehD_{RBD}$ WT used for FAST MD analysis in Fig. 3 (RMSD 0.614).

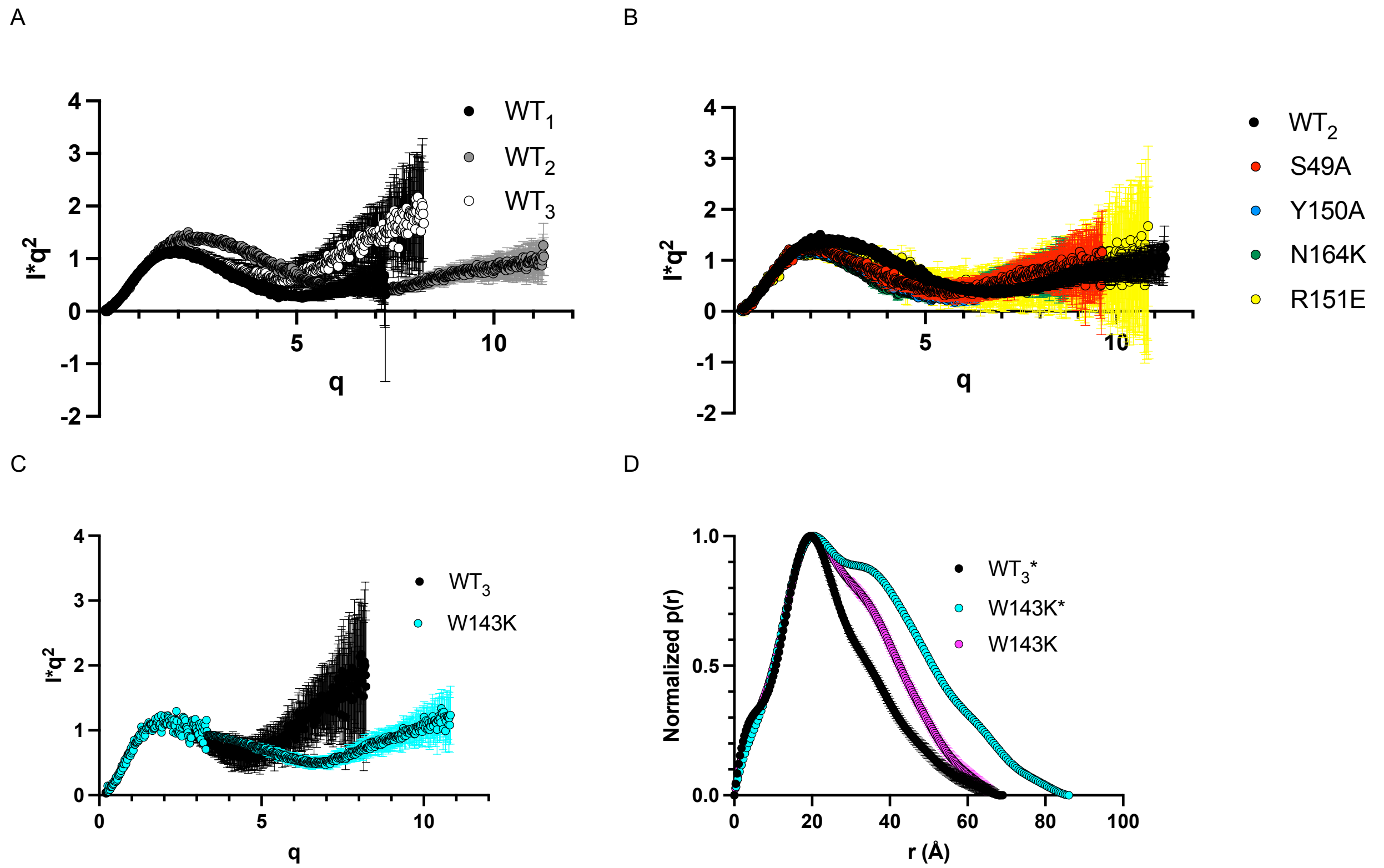


**Fig. S2: Small angle x-ray scattering of $\text{YehD}_{\text{RBD}}$ site-directed mutants. A-C**) Kratky plots of WT replicates (**A**) and site-directed mutants (**B, C**). **D**) Second replicate of W143K (without corresponding WT control) overlain on W143K and its WT control (*) with conserved notching.

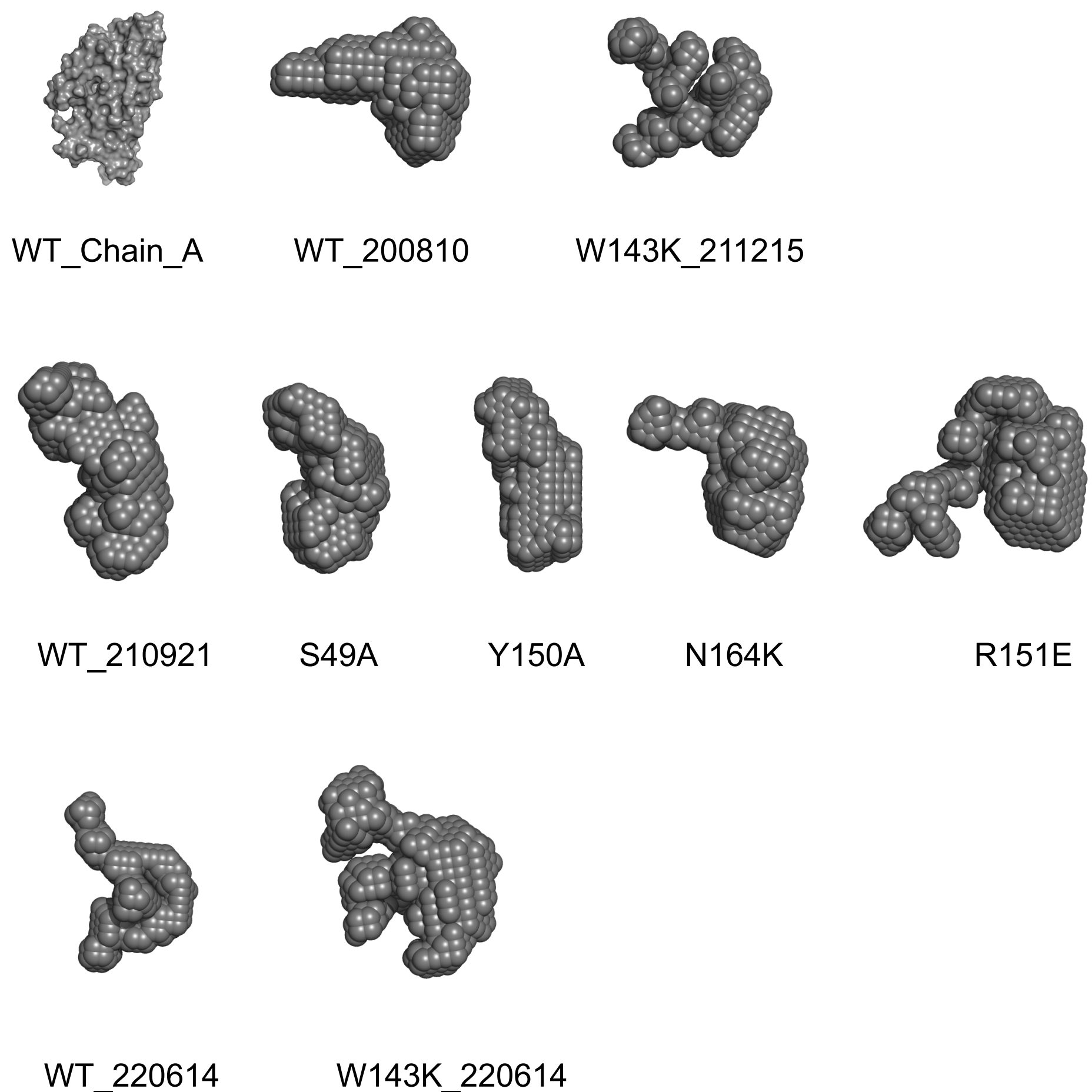


**Fig. S3: *De novo* models of YehD$_{RBD}$ mutants.** Refined models of YehD$_{RBD}$ WT and mutants derived from SAXS shown to scale. Refined models of Damaver/Dammin of 15 separate models shown. Chain A of WT (PDB ID: 9N4G) shown at proximate scale in upper left.

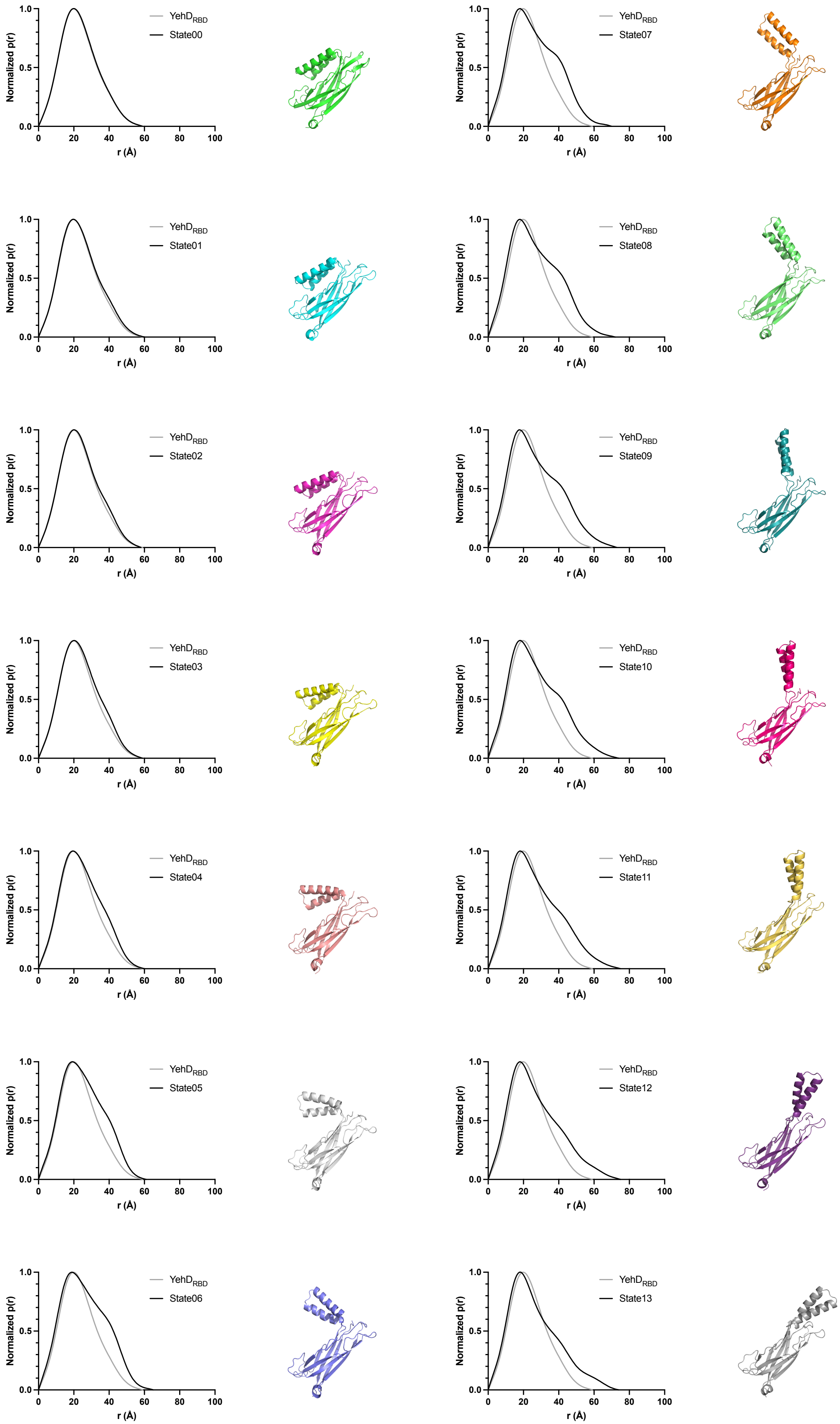

Normalized p(r)
1.0
0.5
0.0
0
20
40
60
80
100
r (Å)
YehD$_{RBD}$
State00
State01
State02
State03
State04
State05
State06
State07
State08
State09
State10
State11
State12
State13

**Fig. S4: CRYSOL generated pairwise [p(r)] functions of intermediate states from FAST modeling.** Left: *In silico* p(r) function of $YehD_{RBD}$ (Chain A, PDB ID: 9N4G) in gray compared to *in silico* p(r) in black of intermediate state. Right: Cartoon model of corresponding intermediate states.

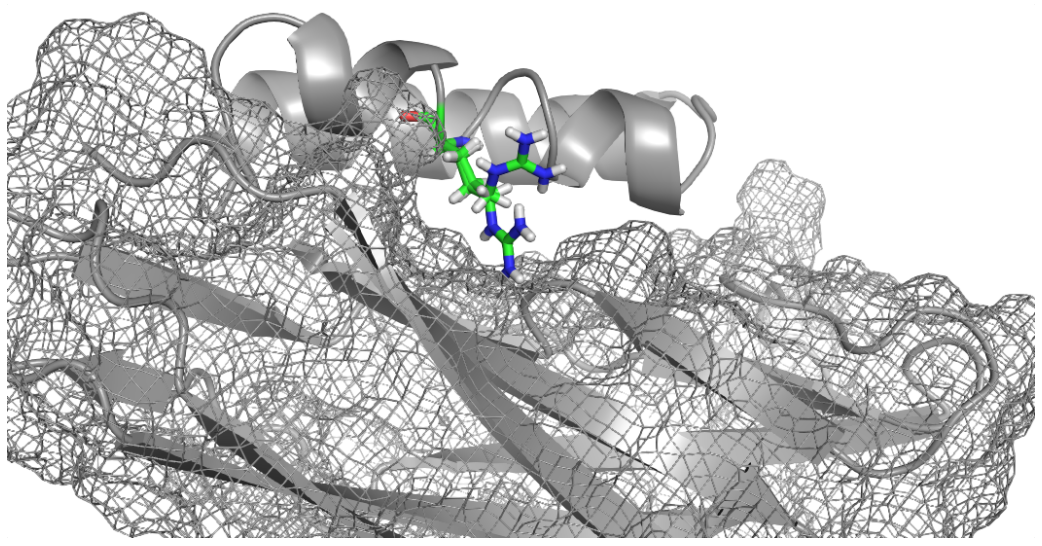

**Fig. S5: Conformations of YehD$_{RBD}$ R151.** Pymol representation of Chain A with both occupants of R151 shown in color. Core surface represented in mesh.

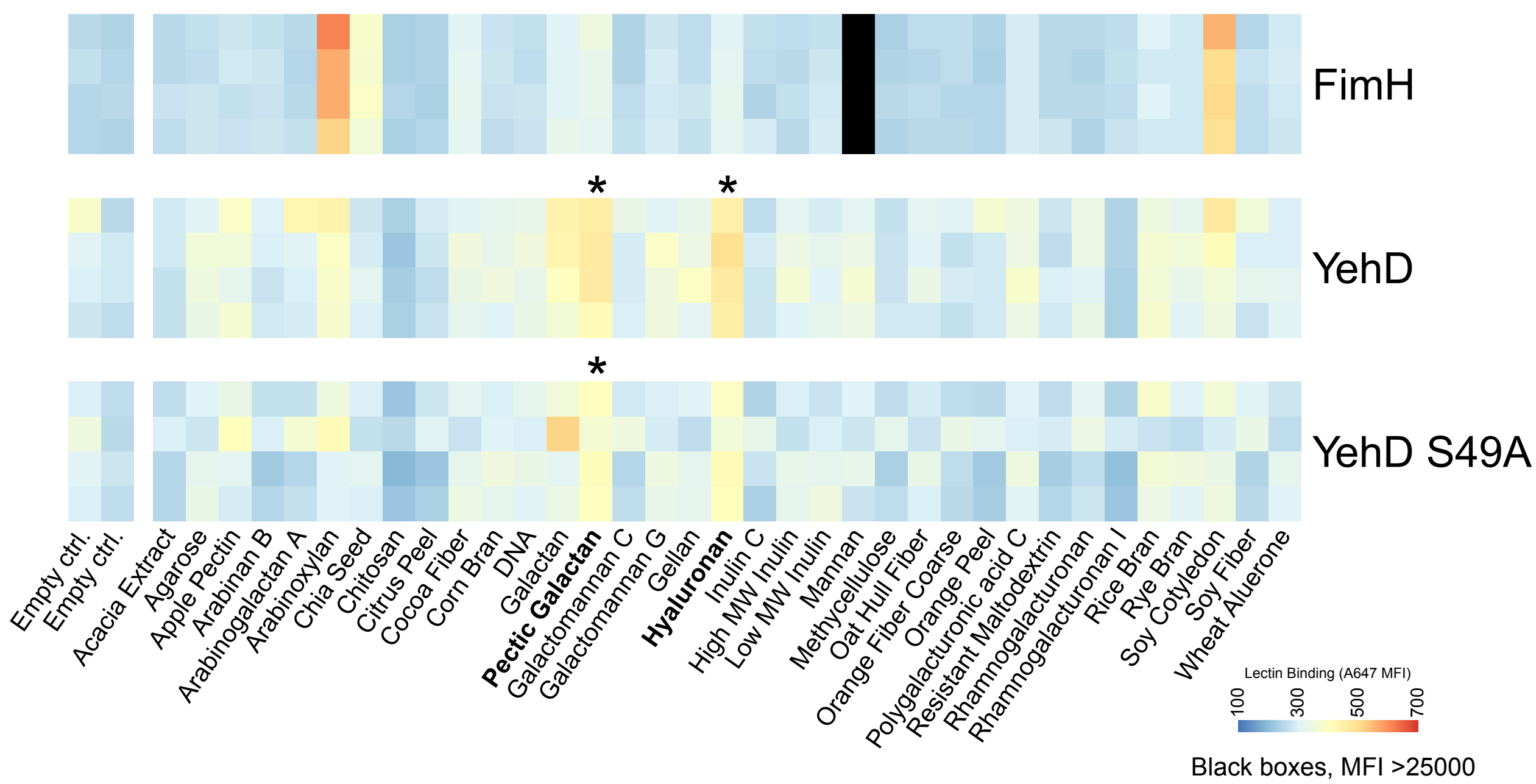


**Fig. S6: Quantification of Alexa-647 labeled YehD$_{RBD}$ WT and S49A binding to glycan coated beads analyzed by flow cytometry**. The geometric mean fluorescence intensity in the Alexa-647 channel for each population of beads is indicated by the color bar. Columns denote the carbohydrate preparation coated on each bead type. Four replicate assays are shown in four rows. Background binding to empty beads was subtracted from the signal for each glycan bead type. Significant differences in binding between each glycan bead versus empty beads are indicated for YehD plots. *, $p<0.05$, t-test, Bonferroni corrected. Mannose binding by FimH was also analyzed as a positive control.

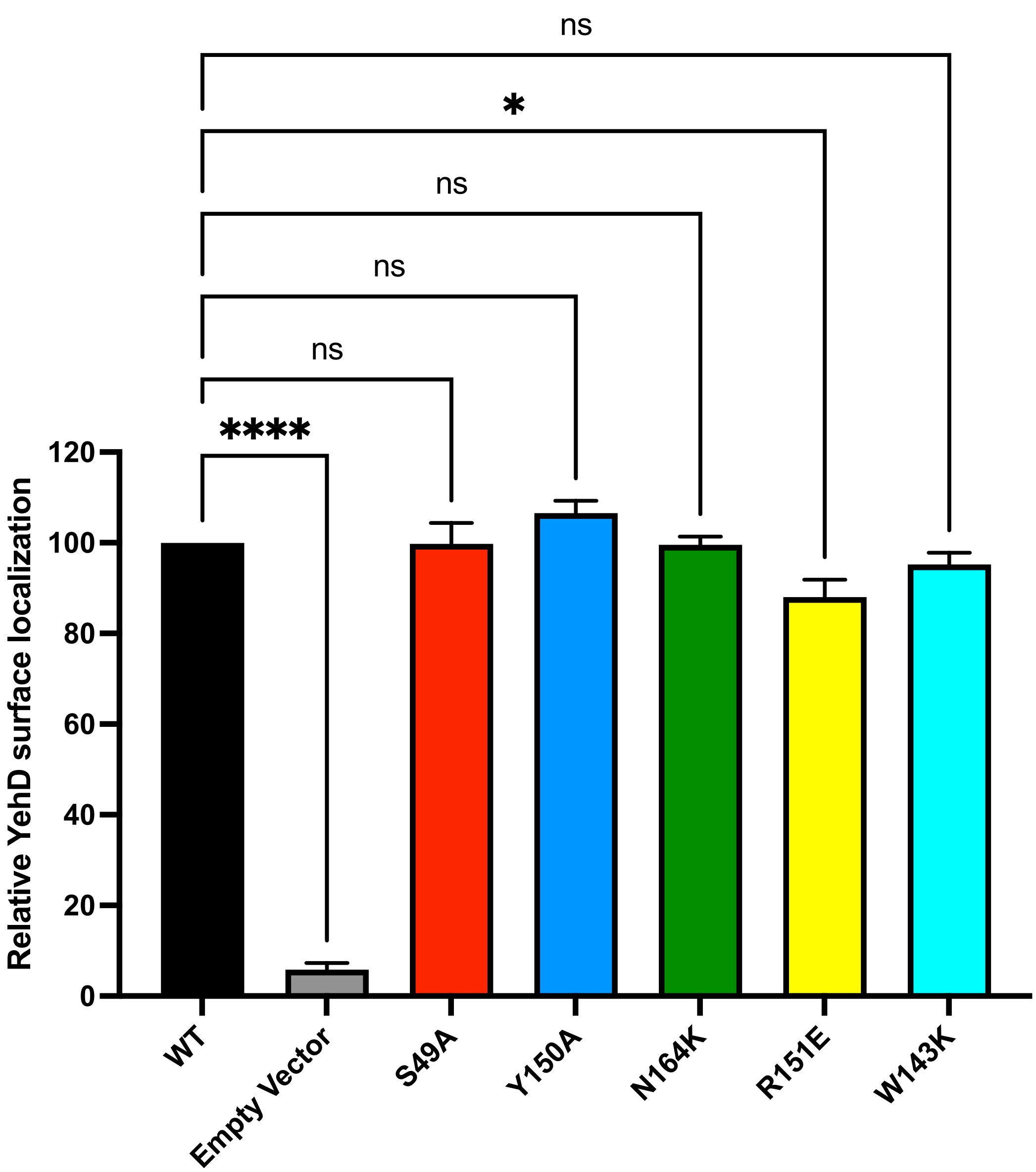


**Fig. S7: Surface expression of YehD mutants.** WT normalized to 100%. Cells expressing mutant YehD proteins atop Yeh pili (expressed from pTRC99a plasmids) were probed with anti-YehD antibodies. One-way ANOVA performed. * - $p < 0.05$, **** - $p < 0.0001$.

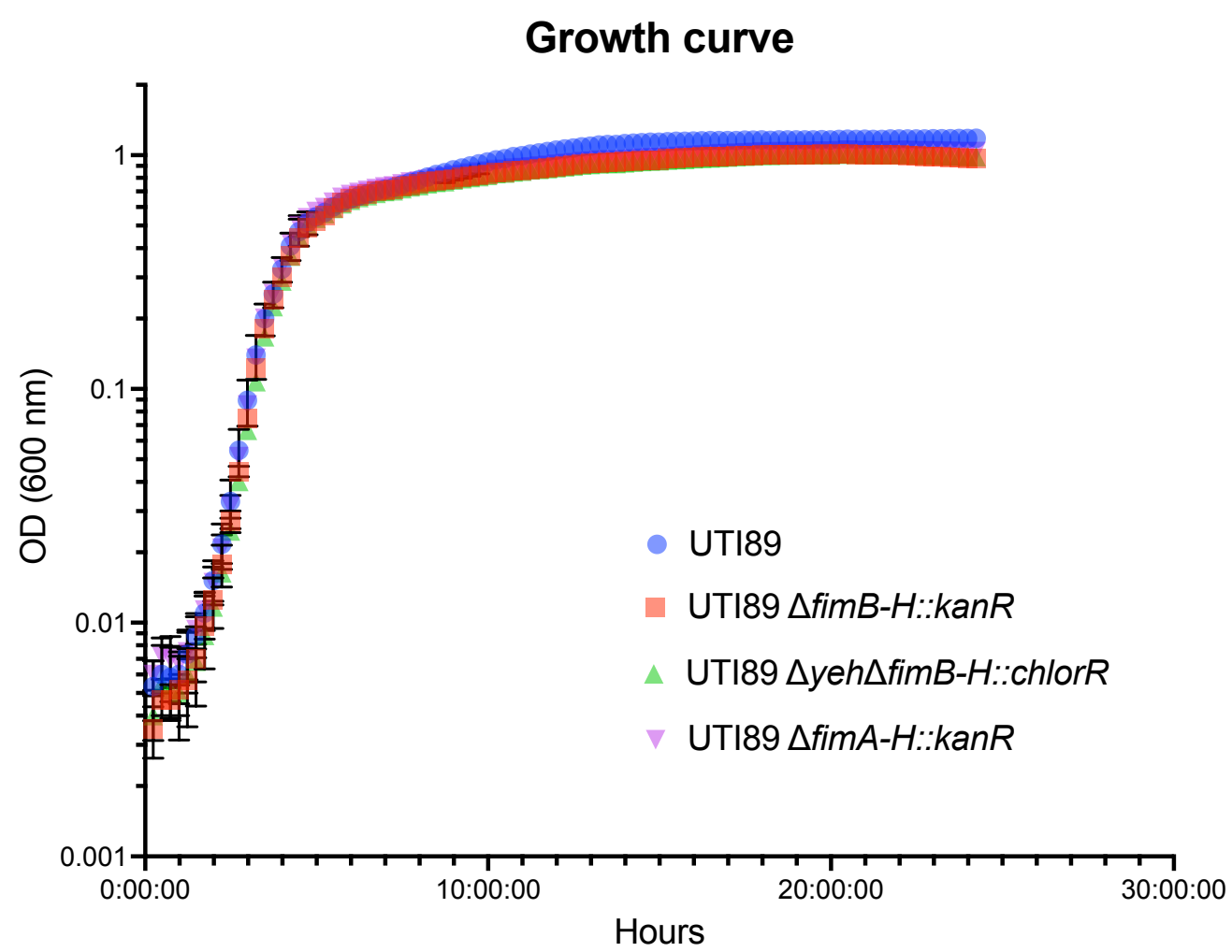


**Fig. S8. Growth curves in LB of strains in competitive colonization experiments.** Standard deviation shown.

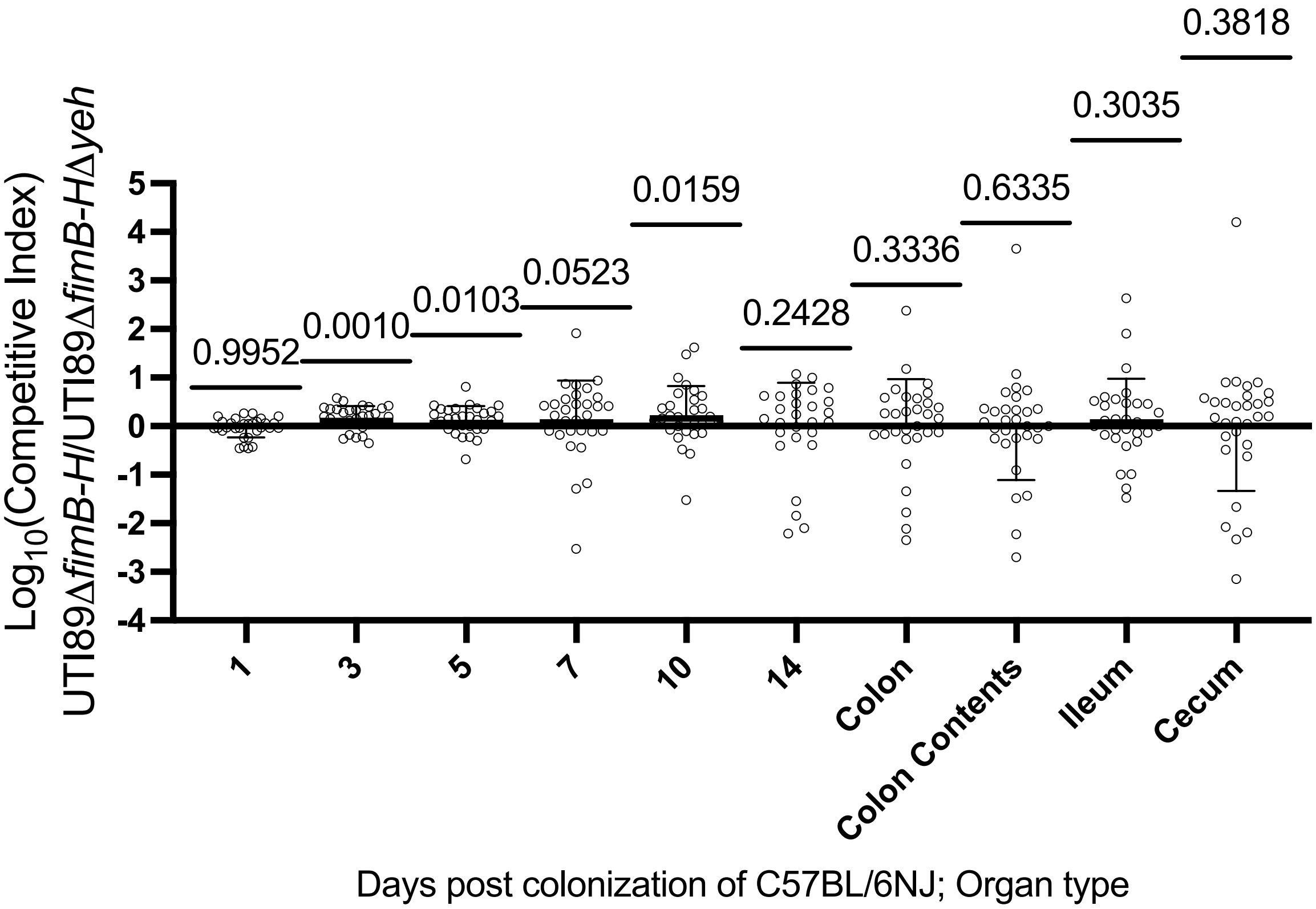


**Fig. S9: Yeh pilus contributes to gastrointestinal tract colonization in the absence of the type 1 pilus in C57BL/6NJ mice.** Competitive indices of individual titers of the competitive colonization experiment competing UTI89Δ*fimB-H* vs. UTI89Δ*fimB-H*Δ*yeh.* N = 30 mice over 2 separate experiments. Log(Competitive Index) of UTI89Δ*fimB-H* vs. UTI89Δ*fimB-H*Δ*yeh* for CFU/g feces in each mouse individually on each day of sampling and Log(Competitive Index) of UTI89Δ*fimB-H* vs. UTI89Δ*fimB-H*Δ*yeh* for CFU/g organ within each mouse on Day 14. Wilcoxon test (including the method of Pratt) was used to determine statistical significance. P values shown above each dataset.
